# Rhamnolipid Self-Aggregation in Aqueous Media: A Long Journey toward the Definition of Structure–Property Relationships

**DOI:** 10.3390/ijms24065395

**Published:** 2023-03-11

**Authors:** Rodolfo Esposito, Immacolata Speciale, Cristina De Castro, Gerardino D’Errico, Irene Russo Krauss

**Affiliations:** 1 Department of Chemical Sciences, University of Naples Federico II, I-80126 Naples, Italy; 2 CSGI (Consorzio per lo Sviluppo dei Sistemi a Grande Interfase), I-50019 Florence, Italy; 3 Department of Agricultural Sciences, University of Naples Federico II, I-80055 Portici, Italy

**Keywords:** biosurfactant, rhamnolipid, physical chemistry, aggregation, surface tension, micellization, interface

## Abstract

The need to protect human and environmental health and avoid the widespread use of substances obtained from nonrenewable sources is steering research toward the discovery and development of new molecules characterized by high biocompatibility and biodegradability. Due to their very widespread use, a class of substances for which this need is particularly urgent is that of surfactants. In this respect, an attractive and promising alternative to commonly used synthetic surfactants is represented by so-called biosurfactants, amphiphiles naturally derived from microorganisms. One of the best-known families of biosurfactants is that of rhamnolipids, which are glycolipids with a headgroup formed by one or two rhamnose units. Great scientific and technological effort has been devoted to optimization of their production processes, as well as their physicochemical characterization. However, a conclusive structure–function relationship is far from being defined. In this review, we aim to move a step forward in this direction, by presenting a comprehensive and unified discussion of physicochemical properties of rhamnolipids as a function of solution conditions and rhamnolipid structure. We also discuss still unresolved issues that deserve further investigation in the future, to allow the replacement of conventional surfactants with rhamnolipids.

## 1. Introduction

In the last few years, increasing attention toward environmental issues is steering the scientific and technological research. The need to protect human and environmental health and to avoid the widespread use of substances obtained from nonrenewable sources has prompted research toward the discovery and development of new sustainable molecules characterized by high biocompatibility and biodegradability, obtained from a natural origin or a synthetic route based on green chemistry principles. A class of substances for which this need is particularly urgent is that of surfactants [1,2,3,4]. Indeed, surface-active agents, whose contraction gives origin to the word surfactants, find large use in an incredible variegate range of applications [5,6]; they are at the basis of all detergent formulations, including those for household [7] and personal care [8], for example; they are amply used in many industrial processes [9,10,11,12,13,14,15], as well as in agriculture [16], oil recovery [11], and remediation processes [17]; they are crucial components of food [18], cosmetic [19], and pharmacological products [19,20,21,22,23,24]. Such a variety of applications and markets justifies surfactant production volumes of about 17 million tons per year [25].

However, both the use and the synthesis of surfactants are associated with unfavorable environmental issues that represent an increasing concern worldwide. Most surfactants are produced from petrochemical resources by means of polluting industrial processes [26,27,28]. Moreover, they can cause allergic reactions and skin irritation [29,30,31] and, when dispersed in the environment, may be toxic to different organisms [25,32,33,34].

For these reasons, the development of environmentally friendly surfactants with good biodegradability is in great demand [35]. In this respect, an attractive and promising alternative to commonly used synthetic surfactants is represented by so-called biosurfactants [1,2,4,19,26,36,37,38]; these are surfactants naturally derived from microorganisms, such as bacteria and yeasts [4,19,26,28,39,40,41]. However, this term is often used in a larger meaning to indicate not only surfactants extracted from natural sources or produced enzymatically or microbially, but also those surfactants that are chemically synthesized from biomass, such as sugars, plant oils, and amino acids [27,42]; for the latter class, a more appropriate term should be bioderived or bioinspired surfactants, and they should be discussed as a class of their own [4].

**Figure 1:**
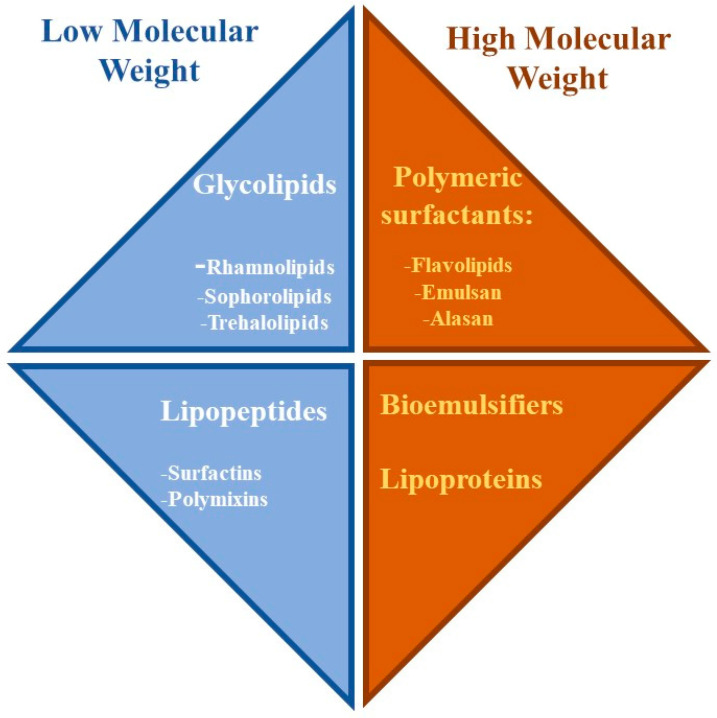
Classification of biosurfactants according to their molecular weight.

Biosurfactants are usually classified on the basis of their molecular weight, a distinction due to Rosenberg and Ron [43], rather than the features of the polar head, as happens for canonical synthetic surfactants; hence, biosurfactants are divided into low-molecular-weight (LMW) and high-molecular-weight (HMW) ones (Figure 1) [2,28,38,43,44,45]. Glycolipids and lipopeptides belong to the former class, while polymeric compounds, such as proteins, polysaccharides, and combined forms of lipoproteins or lipopolysaccharides belong to the latter [2,41,45,46]. Properties of these two kinds of biosurfactants differ one from the other, with HMW biosurfactants characterized by good emulsifying and surface adhesion properties and LMW biosurfactants characterized by strong surface and interfacial tension reduction and wetting abilities [36,44,45,47,48]. LMW biosurfactants are good candidates to replace canonical surfactants, also considering their general capacity to be active at low concentrations and to withstand extreme conditions of pH, temperature, and salinity, as well as their high biodegradability, low toxicity, and better environmental compatibility [4,19,26,38,40,45,49,50].

One of the best-known families of LMW biosurfactants is that of rhamnolipids; these are glycolipids formed by one or two rhamnose units acetylated with up to three long-chain hydroxy fatty acids [1,2,3,26,44,45,50,51,52,53,54,55,56].

Rhamnolipids are typically produced by *Pseudomonas aeruginosa* [53,55,57,58,59,60,61,62,63,64,65,66,67,68,69,70,71,72,73,74,75,76], but they have also been isolated from other *Pseudomonas bacteria* [54,55,77,78,79], as well as from bacteria belonging to other families [80,81,82,83,84], classes [85,86,87,88], or even phyla [89,90,91,92,93]. In bacteria, rhamnolipids play many different physiological roles, including solubilization and uptake of hydrophobic nutrients, adhesion to surfaces, formation and maintenance of biofilms, and cell motility [55,94,95,96,97,98].

The structural properties of microbial rhamnolipids depend on environmental and growth conditions [3,51,52,53,54,55,56,60,68,69,70,99,100]. They are generally obtained as complex mixtures of congeners: mono-rhamnolipids and di-rhamnolipids, differing in the number of rhamnose groups present; within each of these two classes, congeners differ in terms of chain length, degree of branching, and unsaturation of the fatty acid chains [98,101]. As an example, rhamnolipids produced by *P. aeruginosa* are reported to be mixtures of about 30 different molecules; by considering all the possible microbial origins, up to 60 different rhamnolipid congeners and homologs have been identified [3,51,52,95,96,98]. As for the growth conditions, for example, it has been found that, when the strain *P. aeruginosa* LBI grows on hydrophilic substrates, such as glycerol and glucose, di-rhamnolipids are mostly obtained, whereas, when it is grown on hydrophobic carbon sources, mono-rhamnolipids are the predominant homologs in the mixture [69].

Given the potential of rhamnolipids as replacements of conventional surfactants, immediately recognized on the basis of their impressive physicochemical properties, a rich research field has been devoted to improvement of their production, with the aim of optimizing large-scale cheap processes [2,3,44,45,54,55,100,102]. Rhamnolipids are produced at a higher level compared with other bacterial biosurfactants; they are biosurfactants with higher yields, with the only exception being glycolipids produced by yeasts [54]. With respect to synthetic surfactants, their cost is still nearly prohibitive, with the cost of synthetic surfactants being 1–3 USD/kg, and that of rhamnolipids being 20–25 USD/kg, depending on the volumetric productivity of rhamnolipid fermentation [52,103,104]; however, these production costs are still the result of an impressive improvement, since, in the past, rhamnolipids were evaluated to be 1000 times as expensive as conventional surfactants [105]. The high cost of rhamnolipid production is mainly due to fermentation and product purification steps [3,26,54,106,107,108]. Some approaches that have achieved lower production costs include the use of inexpensive substrates [69,76,99,103,109,110], overproducing strains [52,54,111,112], metabolic engineering techniques [107,113,114], and effective downstream processes [115,116,117,118]. In addition, it may be possible to overcome limitations of their high costs by focusing on refined applications (medical and pharmaceutical), whereby the benefits of using rhamnolipids can compensate for their costs [52]. On these premises, it should not surprise that the microbial surfactant market was valued at 14.2 million USD in the year 2020, and it is projected to reach a size of 18.7 million USD by 2027, with the rhamnolipid market projected to reach 3.1 million USD [119].

At the same time, an increasing number of applications of rhamnolipids have been discovered and investigated in the last few years, as discussed in numerous papers [2,19,28,39,44,45,49,50,53,100,104,105,120,121,122,123,124,125,126,127,128,129,130,131,132,133,134,135,136,137,138,139,140,141].

Likely due to the difficulty of handling pure products and to the fact that even small changes in the composition of the congeners lead to significant differences in the behavior of rhamnolipids, the study of physicochemical properties of this class of biosurfactants has been somehow overlooked, with respect to research on their production and applications. Numerous reports on the characterization of rhamnolipids in terms of aggregation and surface/interface properties are present in the literature; moreover, quite recently, excellent papers reviewed the physicochemical properties of biosurfactants [4,44,45,122,142], including rhamnolipids. However, what is still lacking, in our opinion, is an in-depth overall analysis of the surface and aggregation behavior of this class of molecules as a function of the pH, the presence of salts or additives, the purity of the rhamnolipid, or the composition of the mixture employed. In this review, we aim to fill this gap, by presenting a comprehensive and unified discussion of the physicochemical properties of rhamnolipids and discussing what we consider still unresolved issues in the physicochemical behavior of rhamnolipids that deserve further investigation in the future.

### Rhamnolipid Structure

Rhamnolipids are glycolipids in which the amphiphilic structure arises from the presence of a hydrophilic head, consisting of one or two L-rhamnose units (Rha) linked by an α-1,2-glycosidic linkage, and a hydrophobic part, composed of one, two, and, in a few cases, three β-hydroxy-fatty acids linked to each other through an ester bond formed between the β-hydroxyl group of the distal (relative to the glycosidic bond) chain with the carboxyl group of the proximal chain [55,143]. In most cases, the carboxyl group of the distal β-hydroxy fatty acid chain remains free; few congeners, however, have this group esterified with a short alkyl group [55]. The high structural diversity of different rhamnolipid molecules, resulting in a large pool of rhamnolipid homologs that approaches 60 structures, arises from the numerous combinations of the number of rhamnose moieties and the nature of the aliphatic chains, which may be saturated, monosaturated, or polyunsaturated and branched, with chain length varying from C_8_ to C_16_ [55,144]. As an example, Table 1 presents the rhamnolipid congeners produced by *Pseudomonas aeruginosa* grown using two different carbon sources: glycerol and soy [145].

As for the stereochemistry of these molecules, the β-hydroxyl groups of fatty acid chains in natural rhamnolipids are strictly present in the R-configuration [146], but non-natural molecules with different configurations have also been studied, to unveil the effect of stereochemistry on rhamnolipid properties [147,148].

Interestingly, the presence of the free carboxylic group makes rhamnolipids pH-sensitive surfactants. The pKa of the carboxylic group was determined to be around 5.5 [71,149,150]; thus, they are expected to behave as nonionic surfactants at acidic pH below 5.5 and as anionic ones at neutral and basic pH; their physicochemical and aggregation properties depend on both the pH and the presence of ions.

The carboxylic group also affects the rhamnolipid structure in a more subtle way; with respect to most surfactants, where a net distinction between polar head and hydrophobic tails can be made, in rhamnolipids, the carboxylic end of the aliphatic chains contributes to the hydrophilic region of the surfactant and should be considered as part of the head [147]. A consequence is that the length of the hydrophobic tail is indeed shorter than expected on the basis of the number of carbon atoms of the hydroxy aliphatic acids forming the molecule. As an example, only seven methylene groups of the decanoic acid most frequently found in rhamnolipids contribute to the hydrophobic tail of the surfactant. Furthermore, considering the relatively hydrophobic character of the ester linkage, rhamnolipids can resemble surfactants with two separated hydrophilic headgroups, such as gemini surfactants or bolaamphiphiles; indeed, Baccile et al. suggested treating biosurfactants as biobolaamphiphiles [4].

In general, the quite complex molecular structure of rhamnolipids distinguishes them from canonical surfactants, resulting in an inherent difficulty to rationalize and predict their physicochemical behavior, but also likely resulting in their impressive properties.

## 2. Surface Properties and Micellization

The main property of biosurfactants is probably their ability to lower surface/interface tension. This ability allows the presence of such molecules to be identified in bacterial media [36,66,151,152,153,154] and is at the basis of biosurfactant use in many different fields, with the surface tension being a parameter of crucial importance in different phenomena, such as adsorption, wetting, and catalysis [4,100]. Moreover, self-aggregation of biosurfactants, with formation of aggregate/solvent interfaces, results in the micellization phenomenon, on which other fundamental applications, such as detergency or contaminant removal, depend [155,156,157].

Upon their addition to water, thanks to their amphiphilic nature, surfactants adsorb at the water–air interface, with hydrophobic tails pointing toward air and hydrophilic headgroups pointing toward water. The result of this preferential adsorption is the reduction in solution surface tension *γ* [4,158]. In thermodynamic terms, the presence of surfactants at the air/water interface reduces the surface free energy per unit area required to create a new surface.

As the surfactant concentration is increased, the surface tension decreases to a critical concentration where no further change is observed; at this concentration, the air/water interface is saturated by surfactant molecules, and the added surfactant molecules self-assemble into ordered aggregates where hydrophobic tails hide inside, while hydrophilic heads face outward, toward the water phase. Such aggregates are known as micelles, and the concentration at which they start to form is called the critical micelle concentration (*cmc*) [15,159,160,161].

The surface tension of surfactant solutions can be determined as a function of surfactant concentration by means of tensiometric titration experiments [162]. These experiments allow the determination not only of the maximum lowering of surface tension 𝛾_𝑚𝑖𝑛_, defining surfactant effectiveness, but also of its *cmc*. Moreover, from the slope of the plot of 𝛾 versus the logarithm of surfactant molar concentration *c*, it is possible to calculate the maximal surface excess concentration *Γ* by means of the Gibbs equation [163,164,165,166]:

(1) $$\Gamma =-\frac{1}{nRT}\frac{d\gamma }{dlnc}\prime \;$$

where *R* is the gas constant, *T* is the absolute temperature, and *n* is a coefficient that takes into account the dissociation of ionic surfactants known as the Gibbs prefactor. In turn, the area occupied by a surfactant molecule at the air/solution interface, *A_min_*, is given by

(2) $$A_{min}=\frac{1}{\Gamma N_{A}},$$

where *N_A_* is the Avogadro number. *A_min_* gives a good idea of the packing of molecules and on their structuring and interactions at the interface.

Surface properties, including 𝛾_𝑚𝑖𝑛_ and *A*_𝑚𝑖𝑛_, as well as self-aggregation features, such as the *cmc*, have been determined for a great variety of rhamnolipids, for mixtures of congeners and purified forms, for natural rhamnolipids and synthetic ones, and in conditions differing with respect to pH, ionic strength, and presence of additives. In the next sub-sections, these studies are reviewed considering separately complex mixtures of rhamnolipids as derived from microbial fermentation processes (Section 2.1), rhamnolipids presented as pure forms, obtained upon purification of microbial rhamnolipid mixtures, via optimized (bio)synthetic routes (i.e., by knocking down the gene coding for the enzyme responsible for the binding of the second rhamnose unit on mono-rhamnolipids, thus inhibiting production of di-rhamnolipids) (Section 2.2), or rhamnolipids chemically synthesized to investigate the specific effects of acyl chains or stereochemistry (Section 2.3). However, it can also be seen that rhamnolipids presented as pure forms are often not really pure; for example, they are mixture of only mono-rhamnolipids (or di-rhamnolipids) with a largely predominant congener, but the concentration of other molecules differing for the acyl chains is non-negligible. Particularly for “pure” rhamnolipids, we focus on the effects of different conditions, namely, pH and ionic strength, on their properties. Lastly, we discuss the results obtained by means of molecular dynamics on the behavior of rhamnolipids at the air/water interface (Section 2.4).

### 2.1. Crude Extracts

The surface properties of crude extracts obtained by bacterial growth have been amply characterized as the first step in the identification and possible exploitation of rhamnolipids for different applications. We review their surface properties in Table 2, reporting the bacterial strain, the growth conditions, the main congeners present in each mixture, and the *cmc* and *γ_min_* values. For these surfactant mixtures, as it is not possible to univocally define a molecular weight, *cmc* values are reported in milligrams per liter. Moreover, when possible, we report the conditions of pH, temperature, and ionic strength to which rhamnolipids were subjected to prior to the analysis.

Mixtures of rhamnolipids obtained by different bacterial strains and in different growth conditions are characterized by different abilities to lower surface tension, with *γ_min_* values in the range 26–39 mN/m [67,69,73,99]. They are also characterized by a large variety of *cmc* values, ranging from about 10 [64,110] to about 600 mg/L [74]. In particular, the *cmc* depends on the growth medium employed for rhamnolipid production; when sources containing long carbon chains are used, lower *cmc* values are obtained, with respect to cases where glucose or other carbon sources are employed.

Interestingly, an extensive characterization of rhamnolipid mixture obtained from different strains of *P. aeruginosa* (NCIM 5514 [67], BS20 [64], and SG [70]) and from *P. stutzeri* Rhl [68] subjected to different, extreme, conditions of temperature (from 4 to 120 °C), pH (from 2 to 13), and ionic strength (from 0 to 20% *w*/*v* NaCl) highlighted the outstanding resistance of biosurfactants and the possibility to employ them in industrial applications where canonical surfactants undergo degradation and inactivation. For example, almost no effect on surface tension reduction ability was observed upon 1 or 25 h incubation at 100 °C for rhamnolipids from *P. aeruginosa* NCIM 5514 [67] or upon autoclaving at 121 °C for 10 min for rhamnolipids from *P. aeruginosa* BS20 [64]. A decrease but not an abolition of activity, with *γ_min_* values of about 40 mN/m, was found for rhamnolipids from *P. aeruginosa* NCIM 5514 upon incubation at high temperature for 120 h [67]. All kinds of rhamnolipids preserve their activity in a very large pH range, from 2 to 10, and they are also scarcely affected by ionic strength, being tolerant to concentrations of NaCl up to 18% *w*/*v* [64,67,68]. Rhamnolipids produced by *P. aeruginosa* SG in aerobic or anaerobic conditions still reduced the surface tension of water to lower than 31 and 35.0 mN/m, respectively, after treatment at various temperatures (4–121 °C), pH values (2–10), and concentrations of NaCl (0–150 g/L), whereas, when untreated, their *γ_min_* values were 29 and 33 mN/m, respectively [70]. In this respect, it is worth noting that chemical surfactants are generally deactivated by 2–3% salt concentration [36]. Therefore, these findings prove the advantages of rhamnolipid use.

Interestingly another effect of growth conditions was highlighted by Zhao et al., who analyzed the effects of oxygen on rhamnolipid production, by performing bacterial growth in either aerobic or anaerobic conditions [70]. They found that rhamnolipids obtained in aerobic conditions are characterized by lower *cmc* and *γ_min_* values than those obtained in the absence of oxygen. These differences were ascribed to different compositions of rhamnolipid mixtures; aerobically produced rhamnolipids contained fewer mono-rhamnolipids (54.8%) than those produced in anaerobic conditions (94.7%). Specifically, the main congeners were Rha-C_8_-C_10_, Rha-Rha-C_10_-C_12:1_ and Rha-Rha-C_8_-C_10_ for the former and Rha-C_10_-C_12_ and Rha-C_10_-C_10_ for the latter [70]. Given the supposed higher hydrophobicity of mono-rhamnolipids with respect to di-rhamnolipids, it is surprising that aerobically produced rhamnolipids are more surface-active than anaerobically produced ones, but we should not overlook the effects of hydrophobic tails and, most importantly, the fact that we are discussing complex mixtures and not pure products.

Indeed, analysis of Table 2 gives precious insights into the behavior of rhamnolipids produced by different bacteria and in different conditions; however, since it focuses on complex mixtures of different congeners, it does not allow a proper structure–function relationship to be deduced. For this aim to be achieved, it is necessary to move our attention toward the study of rhamnolipids in as pure a form as possible.

### 2.2. “Pure” Rhamnolipids

The surface properties of pure rhamnolipids are reported in Table 3. The capacity to decrease surface tension strongly depends on the surfactant molecular structure, which affects the ability of these molecules to pack at interface, to be dispersed in water, and to arrange in micellar aggregates. Each physicochemical parameter able to affect surfactant molecular structure and reciprocal interactions affects, in turn, the surface activity and aggregation properties of the surfactant. In this respect, in the case of rhamnolipids, the dissociation of the carboxylic group is expected to significantly change their properties, making, in principle, the pH of the solution or the ionic strength crucial parameters, particularly at pH values where the surfactant is expected to be in its anionic form. Thus, we explicitly report the pH at which measurements were performed, as well as if this pH was adjusted by means of addition of buffers or by use of very small quantities of NaOH and HCl solutions, as well as the presence of salts, usually sodium chloride, to increase ionic strength of the solution. For purified rhamnolipids, where it is possible to define a molecular weight, *cmc* values are reported as millimolar concentrations. Moreover, in many cases, it was possible to calculate *A_min_*, as described above. In view of the importance of dissociation to define the properties of ionic surfactants, we also report the value of prefactor *n* used by each author in the Gibbs equation (Equation (1)). It should be 1 for non-dissociable surfactants and in the case of high ionic strengths, while it should be 2 for completely dissociated surfactants [163,164,165]. However, for incompletely dissociated surfactants, at low ionic strength, intermediate values should be considered; thus, the choice of the *n* value is not always straightforward, and abnormal *A_min_* values may be obtained if the choice of *n* was wrong.

**Figure 2:**
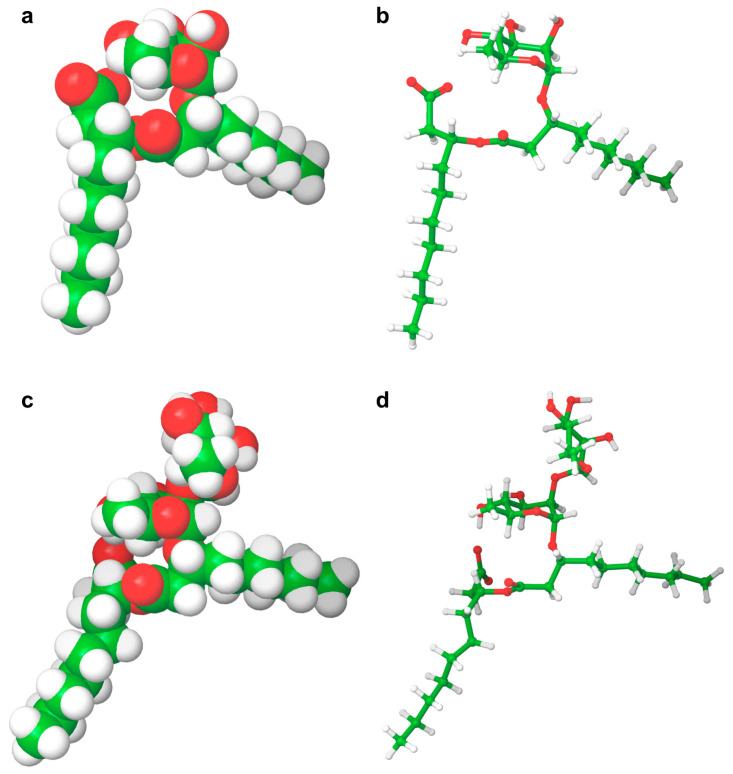
Molecular structures of the most abundant and frequently found rhamnolipids: the mono-rhamnolipid l-rhamnosyl-3-hydroxydecanoyl-3-hydroxydecanoate (Rha-C_10_-C_10_, R1) in (**a**) sphere (CPK) and (**b**) ball & stick representations, respectively, and the di-rhamnolipid α-l-rhamnopyranosyl-α-l-rhamnopyranosyl-β-hydroxydecanoyl-β-hydroxydecanoate di-rhamnolipid (Rha-Rha-C_10_-C_10_, R2) in (**c**) sphere (CPK) and (**d**) ball & stick representations, respectively. The molecules are coloured according to the Atom Type of MacroModel (green: carbon atoms; white: hydrogens; red: oxygen atoms). The structures were built using the Built facility in Maestro program in the Schrödinger suite (Maestro, Schrödinger, LLC, New York, NY, USA) and, then, minimized using AMBER* force field and water as solvent [181].

As can be seen in Table 3, most of the studies concerning pure rhamnolipids focused on the two most abundant and frequently found molecules in complex mixtures of microbial congeners: l-rhamnosyl-3-hydroxydecanoyl-3-hydroxydecanoate (Rha-C_10_-C_10_ or R1) and α-l-rhamnopyranosyl-α-l-rhamnopyranosyl-β-hydroxydecanoyl-β-hydroxydecanoate (Rha-Rha-C_10_-C_10_ or R2), sharing the same composition of the hydrophobic portion and differing in the number of rhamnose units in the polar head (Figure 2).

In this respect, a thorough comparison of the properties of these two representatives of mono- and di-rhamnolipids should shed light on the role of the rhamnose moieties in determining rhamnolipid properties. According to the molecular structures of R1 and R2 only, one should expect R1, being more hydrophobic than R2, to be characterized by lower *cmc* and 𝛾_𝑚𝑖𝑛_ values than R2, while the bulky di-rhamnose headgroup should determine for R2 larger *A_min_* values than for R1. However, analysis of Table 3 clearly indicates that these predictions are not always fulfilled, and that the behavior of this kind of biosurfactants is more complex than expected, as discussed below.

#### 2.2.1. Critical Micellar Concentration

Most of the *cmc* values reported for pure rhamnolipids are determined by means of surface tension titration measurements, and, when other techniques were employed as control, a good agreement among these values was found [35].

For both R1 and R2, different values of *cmc* were determined depending on pH, ionic strength, and presence of additives; *cmc* values range from 0.01 to 0.40 mM in the case of R1, and from 0.01 to 0.46 mM in the case of R2. The very similar *cmc* ranges immediately highlight a close similarity between R1 and R2 despite the different number of rhamnose moieties in the headgroup. For both R1 and R2, the smallest *cmc* values are determined in acidic conditions, pH ~4–5 < 5.6 = pKa, corresponding to conditions where rhamnolipids are expected to be fully protonated, with no net charge and minimal electrostatic repulsions. At acid pH, *cmc* values for R1 and R2 are perfectly coincident when highly pure (>96%) forms of rhamnolipids are used, and the pH is kept constant by the use of buffers [171]. At pH > pKa, *cmc* values of both rhamnolipids significantly increase, in agreement with the supposition that carboxylic groups are deprotonated and the rhamnolipids bear a net negative charge, causing electrostatic repulsion among headgroups. Notably, when highly pure R1 and R2 forms are analyzed in strongly basic conditions (pH = 9) with the pH controlled by means of buffers, R1 is characterized by a *cmc* value that is twice that of R2 [173,174], suggesting that, for rhamnolipids in anionic form, a slight effect of the number of rhamnose moieties in the headgroup could exist.

These results may be rationalized taking into account the contribution of the different intermolecular forces in the micellization process; micellization is essentially an entropy-driven process due to the repulsions between water and the hydrophobic portion of the surfactant. R1 and R2, as said, share the same composition of the hydrophobic part; thus, when no other strong intermolecular force exists (i.e., at very acidic pHs when polar headgroups are not charged and electrostatic interactions are absent), they behave in the same way. On the other hand, when the carboxylic group is negatively charged, increased headgroup hydrophilicity and/or electrostatic repulsion among neighboring headgroups hamper micellization, causing a significant increase in the *cmc*. However, in this case, the second rhamnose unit in the polar headgroup is suggested to be somehow able to partially screen the negative charge [171], resulting in a lower *cmc* for R2 than for R1.

The effect of ionic strength was investigated for both R1 and R2 at acidic [59,71] and neutral [59,71,172,175] pH, when they are supposed to be nonionic and anionic, respectively. It was observed that addition of increasing quantities of NaCl has near no effect when R1 and R2 are protonated. However, it should be noted that experiments at acid pH were performed in the presence of 100 mM acetate buffer, providing a sufficiently high salt concentration that could mask NaCl effects. Moreover, it is known that buffers are able to significantly affect *cmc* values of surfactants even at very low concentrations [182]. On the other hand, by increasing NaCl concentration at pH around 7, *cmc* values decrease for both rhamnolipids, with *cmc* decreasing by a factor of 2–4 for R1 and of 4–5 for R2 [59,71,172,175]. Such a trend agrees with the expected ionic nature of rhamnolipids at neutral pH, with an increase in ionic strength able to better screen the repulsion among charged headgroups brought together in the micellization process, favoring the formation of micelles at lower concentrations than when no salt is present. However, it is worth noting that the variations found for rhamnolipids are small compared to those observed for canonical anionic surfactants; as an example, in the case of sodium dodecyl sulfate, the addition of 25 mM NaCl causes a *cmc* decrease by a factor of 2–8, while addition of 800 mM NaCl causes a decrease by a factor of about 30 [183,184]. Such findings suggest that rhamnolipids have a very slight anionic character and mostly behave as nonionic surfactants.

The *cmc* decrease observed for R1 and R2 is not linear with NaCl concentration and, moreover, follows a different trend for the two rhamnolipids. These results were justified by Helvacy et al., proposing the formation of different kinds of aggregates depending on the nature of the rhamnolipid and the concentration of NaCl [172], but their hypothesis did not find support by other researchers.

Through a thorough comparison of *cmc* values determined for R1 and R2, no clear trend emerges, with R2 *cmc* values higher than or equal to R1 ones, opposite to what is expected on the basis of molecular structures. This is particularly true at neutral–slightly basic pH, as well as when R1 and R2 are the main components of the respective mixtures but other congeners are also present [59,71]. In these cases, it must be recalled that the presence of several congeners, even if in small concentrations, can significantly affect the aggregation of rhamnolipids; for example, the heterogeneity of di-rhamnolipid solutions may cause the formation of aggregates below the classical *cmc* determined by surface tension measurements [178].

Overall, the presence of one or two rhamnose moieties in the headgroup has a slight influence on the micellization of rhamnolipids only when they are in their anionic form. On the other hand, pH affects the *cmc* of rhamnolipids, and an effect of electrolytes is also envisaged at pH > pKa, but much less strong than that found for canonical ionic surfactants.

#### 2.2.2. Surface Tension Reduction

The ability to lower the surface tension of water has been tested for both R1 and R2 in many different conditions, allowing a thorough analysis of their surface behavior to be carried out.

With *γ_min_* values that fall in the 25–36 mN/m range for R1 and in the 28–37 mN/m range for R2, the more hydrophobic R1 appears only slightly more surface-active than the more hydrophilic R2. The highest values are found for both rhamnolipids at highly basic pH when significant dissociation might occur [71,175,176]. As for the smallest values, they are generally found for acidic pH when rhamnolipids fully behave as nonionic surfactants [171]. However, a strict comparison between R1 and R2, as well as a definition of a universal trend of *γ_min_* changes with the pH, is hampered by the presence of congeners in many of the systems analyzed. When highly pure molecules are used, at pH = 5, R1 and R2 are characterized by the same minimum surface tension of 28.2 mN/m [171]. Thus, in the case of pure rhamnolipids, neither the minimum surface tension at the *cmc* nor the value of the *cmc* itself appears to be significantly affected by the rhamnolipid type and depend only on the pH of the solution.

The effect of electrolytes in conditions where R1 and R2 are expected to be deprotonated, pH 6.8, was investigated by Helvacy et al. [172] and Wu et al. [170]. Both research groups found almost irrelevant changes of *γ_min_* for both rhamnolipids up to 1.7 M NaCl. Unfortunately, other studies dealing with the effect of electrolytes at different pHs focused on the *cmc* only and did not determine *γ_min_* as a function of NaCl concentration [59,71]. The minimal change in surface tension observed for R2 with increasing ionic strength was justified by Helvacy et al., invoking its more hydrophilic character with respect to R1 [172]. Moreover, as already stated, the presence of a second rhamnose unit in the polar head was suggested to effectively screen the negative charge, and this could be the cause of R2′s scarce susceptibility to the ionic strength. However, the variation of *γ_min_* found for both R1 and R2 is so small that it could question the actual deprotonation of R1 and R2 at pH 6.8.

#### 2.2.3. Surface Adsorption

For what concerns *A_min_* values, they crucially depend on the choice of the prefactor *n* employed in the Gibbs equation (Equation (1)) [163,164,165,185,186].

At pH < pKa, as well as at pH > pKa and high ionic strength, a Gibbs prefactor *n* = 1 is used, as can be seen in Table 3. In these conditions, *A_min_* values for R1 range between 52 [175] and 84 [172] Å^2^, a significant variation that cannot be related to the different experimental conditions, since the lowest value is found for pure R1 in water at neutral pH and in the presence of 0.5 M NaCl [175] and the highest value is found in very similar conditions, i.e., in pure water, where the pH should be around 7, and in the presence of 1 M NaCl [172]. The main difference is the purity of the rhamnolipid employed: around 96% in the former case, but significantly lower, even if not specified, in the latter case. When only highly pure R1 samples are taken into account, the smallest *A_min_* value, about 60 Å^2^, is found at acidic pH, when electrostatic repulsion among the headgroups should be null [179].

In the case of R2, *A_min_* values range between 65 [171] and 84 [175] Å^2^ when a Gibbs prefactor of 1 is used. Surprisingly the highest value, found in pure water and in the absence of salts, is the same as found for R1, and, overall, *A_min_* values for R2 are not so different or always larger than those found for R1, despite the presence of two rhamnose units in the headgroup rather than one.

At pH > pKa and at low ionic strength, a Gibbs prefactor of 2 should be used to take into account surfactant dissociation [163,164,165,185,186]. In these conditions, *A_min_* for R2 is about 130 Å^2^ [170,171,172,173], while, for R1, it ranges between 86 [174] and 135 [175] Å^2^, a great variability that cannot be easily justified on the basis of the pH or the ionic strength; for example, *A_min_* varies from 86 to 109 Å^2^ by decreasing the pH from 8 to 7 and increasing the phosphate buffer concentration from 10 mM to about 100 mM [174]. Molecular dynamics simulations of anionic Rha-C_10_-C_10_ at the air/water interface indicated an energetically preferred *A_min_* value of ∼80 Å^2^ [187]. However, values ranging from 60 to 140 Å^2^ were all found to be energetically accessible at room temperature. The highest surface concentrations, corresponding to the smallest surface areas, were found in the simulations when the monolayer began to exhibit significant undulations, which depended, in turn, on the balance of possible headgroup–headgroup and tail–tail interactions, which are difficult to predict [187]. This may justify variations of *A_min_* values that cannot be easily linked to molecular structure or solution conditions.

When pure R1 and R2 are analyzed in the same conditions, e.g., in pure water at pH = 6.8 [171,172,173], R1 is characterized by a slightly larger area per molecule at interface, 135 vs. 131 Å^2^. This counterintuitive finding was explained invoking the screening effect of the double rhamnosyl group of R2 molecules on the carboxylate group, allowing R2 molecules to pack more densely than R1 ones at the interface [171], However, such a difference is indeed small and mostly suggests a very similar packing for R1 and R2, irrespective of the supposed bulkiness of the polar head.

As for the effect of ionic strength, Chen et al. reported no change in *A_min_* for R1 and only a slight decrease for R2 in pure water (pH above the pKa) upon addition of 0.5 M NaCl [175], while Helvacy and Ozdemir discussed a doubling of surface concentration and, therefore, a sharp decrease of the area per molecule at the interface, upon addition of 0.05 M NaCl to water solutions of R1 and R2 at pH 6.8 [172]. In this respect, it should be noted that the authors calculated surface concentrations and, consequently, *A_min_* by using a Gibbs prefactor of 2 in the absence of NaCl and of 1 in the presence of NaCl [172]; therefore, the strong variation they observed is likely to have been an artefact of the choice of the Gibbs prefactor with a consequent overestimation of the dissociation of the carboxylate group at pH 6.8 and of the effect of the electrolyte. Indeed, when they further increased the ionic strength but calculated *A_min_* using a prefactor equal to 1, only minimal changes in *A_min_* and no clear trend were observed, with *A_min_* that decreased at 0.5 M and increased at 1 M NaCl in the case of R1, but increased with NaCl concentration for R2, never reaching the values obtained in the absence of salt and with a prefactor of 2 [172].

The structural characterization of interface layers of rhamnolipids by means of different experimental techniques questions the values of *A_min_* determined by means of surface tension titration and Gibbs fitting, particularly the assumption of complete dissociation of the carboxylate group and the use of a Gibbs prefactor of 2. Indeed, investigation of the surface behavior of the mono-rhamnolipid Rha-C_18_-C_18_ by means of polarization modulated-infrared reflection absorption spectroscopy at pH 2, at which the carboxylic acid group of Rha-C_18_-C_18_ should be fully protonated and the molecule is expected to be nonionic, at pH 5, at which the carboxylic acid of Rha-C_18_-C_18_ should be partially deprotonated and Rha-C_18_-C_18_ is expected to be a mixture of nonionic and anionic species, and at pH 8, at which the carboxylic acid should be completely deprotonated, making Rha-C_18_-C_18_ anionic, showed no pH dependence for conformation and packing of molecules in the monolayer [188]. Moreover, complementary investigation of R1 and R2 interface monolayers by means of neutron reflectivity (NR) indicated that there is little systematic difference between the adsorption isotherms measured in water and in pH 9 buffered solutions [175]. Thanks to a strict comparison between the adsorbed amounts of surfactants determined by means of either surface tension or neutron reflectivity experiments, Chen et al. highlighted the necessity to use a Gibbs prefactor greater than 1.0 but smaller than 2.0 to be reconciled, in agreement with the only weakly anionic character of rhamnolipids [176].

### 2.3. Synthetic Rhamnolipids

Apart from the naturally abundant R1 and R2, an extensive characterization of some synthetic rhamnolipids has been reported in the literature, focusing on the effects of stereochemistry [147] and of asymmetry between the acyl chains [148]. Their main physicochemical features are also collected in Table 3.

Natural rhamnolipids have an (R,R) configuration, while Palos Pacheco et al. synthesized the pure (R,S), (S,S), (S,R), and (R,R) diastereomers of R1 and characterized their behavior at both acidic and basic pH (4 and 8, respectively) [147]. The *cmc* values at pH = 4 are very similar for all the diastereomers, with values around 0.015–0.025 mM. It is worth noting that all these values agree well with those determined for natural R1 in acidic conditions and suggest that a mixture of mono-rhamnolipid congeners and even the native mono-rhamnolipid mixture produced by *Pseudomonas aeruginosa* ATCC 9027, an exclusive producer of mono-rhamnolipids, share very similar aggregation properties with the chemically pure (R,R)-Rha-C_10_-C_10_ diastereomer produced by chemical synthesis [147]. The increase in pH to 8 causes a significant increase in *cmc*, with values around 0.2 mM, in agreement with a supposed dissociation of rhamnolipids and with the literature data for natural rhamnolipids, also reported in Table 3 and discussed in Section 2.2. The only exception is represented by the (R,S) diastereomer with a *cmc* of 0.079 mM. Interestingly this diastereomer seems to adopt a structure for which aggregation is more energetically favorable in the anionic form and slightly less favorable in the nonionic form (doubled *cmc* with respect to the other diastereomers at pH = 4) compared to the other diastereoisomers [147].

As for the surface properties, minimum surface tensions of the four diastereomers are all about 28 mN/m, and no significant change is observed with the change in pH, as found also for natural rhamnolipids, indicating that not only the pH but also the lipid tail stereochemistry has little effect on the adsorption of these surfactants at the air/water interface. Similarly, stereochemistry seems to not affect packing at the interface much, with *A_min_* values being very similar for the investigated rhamnolipids at each pH. However, in all cases, a dramatic change in minimum interface area per molecules is found upon changing the pH from 4 to 8. At the latter pH, *A_min_* ranges between 80 and 117 Å^2^; these values are close to those determined for natural R1 at basic pH, when a Gibbs prefactor of 2 is used. The different values obtained for the single diastereomers suggest that slight differences in lipid tail orientation of the surfactants adsorbed at the air/water interface exist, leading to small differences in packing [147]. On the other hand, at pH 4, when rhamnolipids are nonionic and a prefactor of 1 is used, *A_min_* values are almost identical for all diastereomers and very small (about 20 Å^2^). Such surface areas per molecule are smaller than half the values obtained for natural rhamnolipids at acid pH and identical to the cross-sectional area of a single alkyl chain. To rationalize this finding, the authors hypothesized the formation of small lamellar packets of bilayer mono-rhamnolipids, contiguous with the mono-rhamnolipid monolayer at the air/water interface, which results in artificially low values of *A_min_*. Such an arrangement is likely hampered by the presence of different congeners in the natural rhamnolipids analyzed so far [147].

The same researchers also investigated the effect of acyl chain asymmetry by characterizing diastereomeric mixtures of mono-rhamnolipids with the general structure Rha-C_14_-C_x_, where x varies between 6 and 14, as well as the monotailed Rha-C_14_, at pH = 8 [148]. As can be seen in Table 3, the *cmc* significantly changes within the series of Rha-C_14_-C_x_ rhamnolipids, with values ranging between 0.01 and 1.6 mM. According to the increasing hydrophobicity of the surfactant with the lengthening of the second acyl chain, one would expect *cmc* to decrease moving to Rha-C_14_-C_14_; however, experimentally, the authors found different results. Surprisingly, Rha-C_14_-C_14_, despite being more hydrophobic, had a *cmc* higher than Rha-C_14_-C_8_, Rha-C_14_-C_10_, and Rha-C_14_-C_12_, but less than four times lower than the much more hydrophilic Rha-C_14_-C_6_. Minimum surface tension varied much less than *cmc* within the series, with *γ_min_* ranging between 24 and 36 mN/m, but followed the same unexpected trend, with the only exception being the monotailed Rha-C_14_. Notably, Rha-C_14_-C_6_ and Rha-C_14_-C_14_ were characterized by the same minimum surface tension, which was also the highest among the different congeners (36 mN/m). The minimum surface area for molecule was calculated in all cases using a Gibbs prefactor of 2 and, thus, considering the rhamnolipids completely dissociated. The values obtained in this way varied very much, with a minimum value of 38 Å^2^ for Rha-C_14_-C_10_ and a maximum value of 114 Å^2^ for Rha-C_14_-C_6_. As said in Section 2.2.3, such variability could depend on the formation of highly undulated monolayers promoted by intermolecular interactions among headgroups and/or tails of surfactants that are difficult to predict [187]. Another intriguing possibility is that the optimization of interactions between acyl tails, particularly when they have similar length, causes a conformational change that in turn affects the size of the polar headgroup, enhancing or diminishing steric repulsions in an unpredictable way.

### 2.4. Computational Results

Precious insights into the properties of rhamnolipids have also been obtained by means of molecular dynamics (MD) simulations. In particular, the research group of Schwartz and Pemberton investigated the aggregation of R1 in its nonionic and anionic forms [187], as well as the nonionic form of R2 [189] at the air/water interface, while Euston et al. focused on R1 and R2, in both ionic and nonionic forms, and mono-rhamnolipids differing in the length and symmetry of alkyl chains (Rha-C_14_-C_14_, Rha C_14_-C_10_ and Rha C_10_-C_14_) [190] to elucidate the role played by the rhamnose units, the deprotonation of the carboxylate group, and the hydrophobic tails in determining surface properties of these molecules.

At the air/water interface, the presence of the second rhamnose unit determines a slightly different orientation of alkyl chains, but no change in the alkyl chain tilt, a different preferred conformation of the headgroup and the tails (one of the chains is more elongated than the other in R2 but not in R1), and a slight larger surface area per molecule at complete surface coverage (90 vs. 80 Å^2^) for R2 with respect to R1. These differences may be the cause of the different pressure–area isotherms calculated for the two molecules, but they are so small that the surface behavior of R1 and R2 can reasonably be considered the same [189]. Euston et al. investigate using molecular dynamics simulation less packed air/water interfaces than Luft et al. did; nevertheless, they found the same density profiles, overall layer thickness, and distribution of components for adsorbed R1 and R2, and only a 10 Å^2^ difference in surface area per molecule values (70 Å^2^ vs. 60 Å^2^ for R2 and R1, respectively) [190].

MD simulation results agree with experimental NR data indicating that R1 and R2 are characterized by similar reflectivity profiles and the same thickness of the adsorbed monolayer (about 22 Å) [175].

When focusing on R1, Munusamy et al. found that its two forms, anionic and nonionic, present the same area per molecule (80 Å^2^), an equal structure of the monolayer at complete surface coverage concentration, and a similar pressure–area isotherm, indicating an indistinguishable behavior at the air/water interface for charged and non-charged molecules [187]. Such findings are in agreement with the results obtained by MD simulations of R1 and R2 in their charged and nonionic forms by Euston et al.; the average area per molecule at the interface did not change with the charge for both R1 and R2, while an increase of 10 Å^2^ was found for the charged form of congeners with longer tails [190].

A deeper look at R1 molecules at the interface revealed a different hydration pattern and stability of hydrogen bonds for the headgroup of anionic and nonionic forms [187].

Thus, molecular dynamic simulations confirm the identical surface behavior of nonionic and anionic forms of rhamnolipids that also emerges from the analysis of experimental results reported in Table 3, but they are able to highlight slight structural differences between the two forms that could be the cause of this unexpected finding; the rhamnolipid headgroup forms a sort of pocket in which the anionic charge is buried along with its counterion, and this headgroup configuration could isolate the charge and its counterion from neighboring headgroups, minimizing electrostatic repulsion and allowing little to no impact of headgroup charge on packing within the monolayer [188].

MD studies also tried to shed light on the properties of rhamnolipid congeners differing in the hydrophobic region; they showed that, in contrast to the number of rhamnose units and the charge state of the carboxylic group, variations in the hydrophobic region of rhamnolipid molecules, either an increase of the length of the alkyl chains or an asymmetry between tails, have several effects on the properties of these molecules [190]. In particular, increasing the length of the alkyl chains increases the area occupied at the interface, and the different values obtained for Rha-C_10_-C_14_ and Rha-C_14_-C_10_ indicate that the position of the alkyl chains is also important. An asymmetry of the length of alkyl chains causes a different tilt of the second tail, while, in symmetric molecules, they all have similar values. Moreover, mono-rhamnolipids with longer tails (Rha-C_14_-C_14_, Rha-C_14_-C_10_, and Rha-C_10_-C_14_) all present different surface areas for charged and non-charged forms [190]. Thus, it can be stated that there are differences in the conformations adopted by the various rhamnolipids at the air/water interface, but most of these are relatively small; hence, the effect on surface activity may also be small.

Overall, experimental and computational results indicate that the presence of one or two rhamnose units has nearly no effect on the surface properties and micellization of rhamnolipids. A further confirmation of this conclusion comes from the characterization of R1/R2 mixtures, for which an ideal mixing behavior and micellization and surface properties very similar to those of single components is observed [166,173,175].

Quite unexpectedly, the pH, the ionic strength, and the stereochemistry seem to not significantly affect the capability of rhamnolipids to lower the surface tension of water solutions. At a first glance, analysis of Table 3 indicates that the pH affects micellization and apparently the packing of molecules at the air/water interfaces; however, concerning the latter point, great precautions are required when discussing *A_min_* values, since big variations are likely to be artefacts due to the choice of the Gibbs prefactor, as neutron reflectivity, polarization modulated-infrared reflection absorption spectroscopy, and molecular dynamics simulations have pointed toward an identical aggregation of anionic and nonionic forms of rhamnolipids at the air/water interface.

Lastly, the length and, in particular, the symmetry of the acyl chains appear to have a significant effect on the micellization and surface properties of rhamnolipids, but this cannot be easily predicted because experimental and computational data on this subject are still scarce.

## 3. Aggregation Behavior

Glycolipids self-assemble into spherical, disc-like (oblate) or rod-like (prolate) spheroid micelles in dilute conditions [122,191], while, at higher concentrations, they show a complex phase behavior with a range of liquid crystalline states [122,192]. Similarly, rhamnolipids are suggested to form various structures, such as micelles, vesicles, bilayers, and various mesophases, which could be important for their applications. In principle, the presence of the carboxylic acid group further complicates this scenario, conferring a pH dependence to rhamnolipid aggregation properties, in addition to the dependence on concentration, temperature, presence of additives, salts, or impurities, and sample heterogeneity due to the presence of different congeners. Therefore, in Table 4, we report the nature and the dimensions of the aggregates formed by mono- and di-rhamnolipids, as well as the degree of purity, the temperature, the pH, the presence of additives, and the experimental technique employed for analysis of aggregates.

Rhamnolipids are indeed able to form aggregates with very different dimensions, depending on the concentration, the pH, and the temperature. However, a clear identification of the kind of aggregates formed is often not possible as many studies employed dynamic light scattering (DLS) as the only technique to analyze aggregate features, providing only the hydrodynamic radius (*R_h_*) or diameter. In this respect, rhamnolipids are shown to form mainly smaller aggregates with hydrodynamic radii on the order of tens of nanometers (10–60 nm) and larger aggregates with hydrodynamic radii on the order of hundreds of nanometers. A first source of confusion arises because the former aggregates are considered as micelles by some research groups and as vesicles by others. Actually, species with *R_h_* ~10/30 nm are probably micelles; however, because of their large dimensions, they cannot be identified as simple spherical micelles and are likely to be elongated, ellipsoidal, or even cylindrical ones. Aggregates with *R_h_* ~50/60 nm could be identified as vesicles, but a clear distinction between very large micelles and vesicles requires the use of additional experimental techniques in conjugation with DLS, such as small-angle neutron scattering (SANS) or polarized optical microscopy (POM). Nonetheless, it is worth noting that neither of these two techniques gives a clear-cut definition of the aggregate nature on its own. When SANS or POM are employed, the presence of a lamellar arrangement of rhamnolipids is assessed by a q^−2^ trend of the SANS profiles at small *q* or by the presence of Maltese crosses in POM images; however, whether we are in the presence of flat lamellae or spherical vesicles (and if the latter are uni- or multi-lamellar) cannot be established without the combination of complementary techniques. Therefore, the actual nature of lamellar aggregates is also elusive. Lastly, some ambiguous or wrong classifications could be the result of misinterpretation of the experimental data.

The aggregation behavior of R1 and R2 has also been rationalized in some cases through the calculation of the critical packing parameter (*cpp*) [193,194], but very different values are reported in different papers, with *cpp* values ranging from 0.24 [172] to 1 [144] for R1 and from 0.27 [172] to 0.73 [179] for R2.

The *cpp* is defined as

(3) $$cpp=\frac{v_{0}}{l_{0}a_{e}},$$

where $v_{0}$ an $l_{0}$ are the volume and the length of the hydrophobic tail, respectively, and $a_{e}$ is the equilibrium area occupied by the headgroup at the interface [193].

Within the simplicity of the above formula, calculating the *cpp* of surfactants, particularly complex surfactants such as biosurfactants in general and rhamnolipids in particular, is not trivial [4]. Focusing only on the main issues, one has to consider not only the possibility of tail deformation when defining $v_{0}$ and $l_{0}$, but also, in the case of rhamnolipids, the presence of two tails and their actual lengths, since delimitation of hydrophobic and hydrophilic regions is much more blurred than in canonical surfactants. The most difficult geometrical parameter to calculate among those present in the *cpp* definition is $a_{e}$. Often, it is regarded as an index of the steric hindrance of the headgroups; considering this oversimplification, the conformational flexibility of the rhamnolipid headgroup, particularly for di-rhamnolipids, significantly complicates $a_{e}$ evaluation. In addition, $a_{e}$ strictly represents an equilibrium area per molecule, obtained from the minimization of the free energy of micellization. Therefore, it includes effects deriving from electrostatic interaction, hydration of the headgroup, binding and hydration of counterions, and hydrophobicity of the headgroup [4,195], all of which are effects difficult to evaluate and more significant for rhamnolipids than for canonical surfactants. Lastly, the influence of the hydrophobic region usually is not accounted for in $a_{e}$ calculation [194,196], but is likely to play a crucial role in determining headgroup conformation in the case of rhamnolipids. For all these reasons, the observed variability of *cpp* values is not surprising; moreover, great caution is required when ascribing a specific morphology to rhamnolipid aggregates on the basis of *cpp* values.

With these premises, we nonetheless attempt an analysis of rhamnolipid aggregation behavior as a function of the different environmental and structural parameters. We keep the distinction between “pure” (Section 3.1) and synthetic (Section 3.2) rhamnolipids presented in Section 2, while we skip the review of the aggregation behavior of crude extracts obtained by microbial fermentation, which has been carried out in several cases [62,74,197,198], since it is our opinion that it does not significantly contribute to elucidation of structure–function relationships, given the inherent complexity of the behavior of “pure” forms. Lastly, we discuss insights from molecular dynamics simulations (Section 3.3).

### 3.1. “Pure” Rhamnolipids

As can be seen in Table 4, most of the studies dealt with rhamnolipids R1 and R2 in neutral or basic conditions, above their pKa, where they are expected to behave as anionic surfactants; very few data are available on rhamnolipid aggregation behavior in acidic conditions where they have a fully nonionic character.

**Table 4:** Aggregation properties of “pure” rhamnolipids obtained either by purification of microbial extracts or by modified biosynthetic routes. Based on the rhamnolipid pKa = 5.5, rhamnolipids are expected to be neutral at pH < 5.5 and negatively charged at pH > 5.5.

Rhamnolipid	Purity (%)	Concentration (mM)	T	pH	Additive	Aggregate	Dimensions (R, nm)	Experimental Technique ^§^	*cpp*	Ref
Rha-C_10_-C_10_	96	0.02–0.1	25	6.8		Micelles	75 * 25 **	DLS	0.62	[179]
	96	0.5–1	25	6.8		Micelles	80 * 35 **	DLS	0.62	[179]
	96	10	25	6.8		Micelles	100 * 50 **	DLS	0.62	[179]
	96	15–20	25	6.8		Vesicles	30, 125 * 20 **	DLS	0.62	[179]
	96	45	25	6.8		Vesicles	30, 150 * 20 **	DLS	0.62	[179]
	96	1	25	6.8		Elliptical vesicles	75	SEM	0.62	[179]
	98 ^†^	5	RT	8		Ellipsoidal micelles Vesicles	2.2, 10.5, 100 * 2.2 **	DLS	0.48	[174]
	98 ^†^	20	RT	8		Ellipsoidal micelles Vesicles	2.8, 10.2, 90 * 2.8 **	DLS		[174]
	98 ^†^	0.05–15	RT	8	250 nM prodan	Lamellar aggregates		Fluorescence		[174]
	98 ^†^	0.05–15	RT	4	250 nM prodan	Lamellar aggregates		Fluorescence		[174]
	98 ^†^	0.05–15	RT	8	250 nM prodan	Micelles		Fluorescence		[174]
	98 ^†^	10	RT	8	250 nM pyrene	Micelles		Fluorescence		[174]
	96	>5% (*w*/*v*)	RT	6.8	-	Spherical micelles/lamellar		POM	0.24	[172]
	96	>5% (*w*/*v*)	RT	6.8	0.05 M NaCl	Spherical micelles/lamellar		POM	0.50	[172]
	96	>5% (*w*/*v*)	RT	6.8	0.5 M NaCl	Lamellar/bilayers or multilamellar		POM	0.61	[172]
	96	>5% (*w*/*v*)	RT	6.8	1.0 M NaCl	Isotropic hexagonal		POM	0.41	[172]
	Purified	20		9	Borax 0.023 M HCl 0.008 M	Micelles		SANS		[176]
	Purified	30–100		9	Borax 0.023 M HCl 0.008 M	Lamellar structures		SANS		[176]
	Purified	0.02	RT	6.8		Micelles	25	DLS		[170]
	Purified	0.05	RT	6.8		Micelles	28	DLS		[170]
	Purified	0.1	RT	6.8		Micelles	28	DLS		[170]
	Purified	0.5	RT	6.8		Micelles	39	DLS		[170]
	Purified	1.0	RT	6.8		Micelles	45	DLS		[170]
	Purified	0.5	293	6.8		Micelles	29	DLS		[170]
	Purified	0.5	298	6.8		Micelles	25	DLS		[170]
	Purified	0.5	303	6.8		Micelles	26	DLS		[170]
	Purified	0.5	308	6.8		Micelles	34	DLS		[170]
	Purified	0.5	313	6.8		Micelles	30	DLS		[170]
	Purified	0.5	318	6.8		Vesicles	130	DLS		[170]
	Purified	0.5	323	6.8		Vesicles	115	DLS		[170]
	Purified	0.5	RT	2.5		Vesicles	170	DLS		[170]
	Purified	0.5	RT	3.5		Micelles/vesicles	70	DLS		[170]
	Purified	0.5	RT	4.5		Micelles/vesicles	70	DLS		[170]
	Purified	0.5	RT	5.5		Micelles	35	DLS		[170]
	Purified	0.5	RT	6.5		Micelles	38	DLS		[170]
	Purified	0.5	RT	7.5		Micelles	38	DLS		[170]
	Purified	0.5	RT	8.5		Micelles	30	DLS		[170]
	Purified	0.5	RT	9.5		Micelles	25	DLS		[170]
	Purified	0.5	RT	10.5		Micelles	23	DLS		[170]
	Purified	20	RT	9	Borax 0.023 M HCl 0.008 M	Ellipsoidal micelles	1.4 ^a^ 1.7 ^b^ N_agg_ = 47	SANS		[199]
	Purified	20	RT	9	Borax 0.023 M HCl 0.008 M 2 mM Ca^2+^	Ellipsoidal micelles	1.5 ^a^ 1.8 ^b^ N_agg_ = 55	SANS		[199]
	95 ^††^	0.05	RT	7.4	Hepes 5 mM/NaCl 100 mM	Micelles	25	DLS		[71]
	95 ^††^	0.5	RT	7.4	Hepes 5 mM/Na Cl 100 mM	Cylindrical micelles	105	DLS		[71]
	90	20	RT	9	Borax 0.023 M HCl 0.008 M	Ellipsoidal micelles		SANS		[175]
	90	50	RT	9	Borax 0.023 M HCl 0.008 M	Lamellar structures		SANS		[175]
		1	RT	7.17		Vesicles	130	DLS		[149]
		1	RT	3.20		Large aggregates	1250	DLS		[149]
**Rha-Rha-C_10_-C_10_**	99	>5% (*w*/*v*)	RT	6.8	-	Spherical micelles/ lamellar		POM	0.27	[172]
	99	>5% (*w*/*v*)	RT	6.8	0.05 M NaCl	Spherical micelles/ lamellar		POM	0.52	[172]
	99	>5% (*w*/*v*)	RT	6.8	0.5 M NaCl	Lamellar/bilayers or multilamellar		POM	0.52	[172]
	99	>5% (*w*/*v*)	RT	6.8	1.0 M NaCl	Isotropic hexagonal		POM	0.40	[172]
	50% ^†††^	0.125	25	7.4	5 mM Hepes 0.15 M NaCl	Cylindrical micelles Vesicles	20–30 175–275	DLS		[59]
	50% ^†††^	0.25	25	7.4	5 mM Hepes 0.15 M NaCl	Cylindrical micelles Vesicles	20–30 175–275	DLS		[59]
	50% ^†††^	0.5	25	7.4	5 mM Hepes 0.15 M NaCl	Cylindrical micelles Vesicles	20–30 175–275	DLS		[59]
	50% ^†††^	1	25	7.4	5 mM Hepes 0.15 M NaCl	Cylindrical micelles Vesicles	20–30 175–275	DLS		[59]
	50% ^†††^	1.5	25	7.4	5 mM Hepes 0.15 M NaCl	Vesicles	175–275 >750	DLS		[59]
	50% ^†††^	2.5	25	7.4	5 mM Hepes 0.15 M NaCl	Vesicles	>750	DLS		[59]
	50% ^†††^	5	25	7.4	5 mM Hepes 0.15 M NaCl	Vesicles	>750	DLS		[59]
	50% ^†††^	>2.5	25	7.4	5 mM Hepes 0.15 M NaCl	MLV Elongated vesicles	>75–100 >500	TEM		[59]
	50% ^†††^	20	25	7.4	5 mM Hepes 0.15 M NaCl	Lamellar multilayers		SAXS		[59]
	Purified	50	RT	9	Borax 0.023 M 0.008 M HCl	Ellipsoidal micelles	1.15 ^a^ 1.5 ^b^ N_agg_ = 34	SANS		[176]
	Purified	20	RT	9	Borax 0.023 M 0.008 M HCl	Ellipsoidal micelles	1.2 ^a^ 1.5 ^b^ N_agg_ = 26	SANS		[175]
	Purified	50	RT	9	Borax 0.023 M HCl 0.008 M	Ellipsoidal micelles	1.2 ^a^ 1.5 ^b^ N_agg_ = 34	SANS		[175]
	Purified	100	RT	9	Borax 0.023 M 0.008 M HCl	Ellipsoidal micelles	1.1 ^a^ 1.5 ^b^ N_agg_ = 86	SANS		[175]
	Purified	20	RT	9	Borax 0.023 M 0.008 M HCl	Ellipsoidal micelles	1.2 ^a^ 1.5 ^b^ N_agg_ = 26	SANS		[199]
	Purified	20	RT	9	Borax 0.023 M 0.008 M HCl 2 mM Ca^2+^	Ellipsoidal micelles	1.2 ^a^ 1.6 ^b^ N_agg_ = 28	SANS		[199]
	Purified	50	RT	9	Borax 0.023 M HCl 0.008 M	Ellipsoidal micelles	1.2 ^a^ 1.5 ^b^ N_agg_ = 34	SANS		[199]
	Purified	50	RT	9	Borax 0.023 M 0.008 M HCl 2 mM Ca^2^	Ellipsoidal micelles	1.2 ^a^ 1.6 ^b^ N_agg_ = 38	SANS		[199]
	Purified	100	RT	9	Borax 0.023 M HCl 0.008 M	Ellipsoidal micelles	1.1 ^a^ 1.5 ^b^ N_agg_ = 86	SANS		[199]
	Purified	100	RT	9	Borax 0.023 M HCl 0.008 M 2 mM Ca^2^	Ellipsoidal micelles	1.1 ^a^ 1.4 ^b^ N_agg_ = 91	SANS		[199]
	Purified	0.02	RT	6.8		Micelles/vesicles	75	DLS		[170]
	Purified	0.05	RT	6.8		Micelles	35	DLS		[170]
	Purified	0.1	RT	6.8		Micelles	35	DLS		[170]
	Purified	0.5	RT	6.8		Micelles	22	DLS		[170]
	Purified	1.0	RT	6.8		Micelles	30	DLS		[170]
	Purified	0.5	293	6.8		Micelles	25	DLS		[170]
	Purified	0.5	298	6.8		Micelles	30	DLS		[170]
	Purified	0.5	303	6.8		Micelles	25	DLS		[170]
	Purified	0.5	308	6.8		Micelles	20	DLS		[170]
	Purified	0.5	313	6.8		Micelles	15	DLS		[170]
	Purified	0.5	318	6.8		Micelles/vesicles	90	DLS		[170]
	Purified	0.5	323	6.8		Vesicles	95	DLS		[170]
	Purified	0.5	RT	2.5		Vesicles	230	DLS		[170]
	Purified	0.5	RT	3.5		Vesicles	145	DLS		[170]
	Purified	0.5	RT	4.5		Vesicles	140	DLS		[170]
	Purified	0.5	RT	5.5		Micelles/vesicles	80	DLS		[170]
	Purified	0.5	RT	6.5		Micelles/vesicles	70	DLS		[170]
	Purified	0.5	RT	7.5		Micelles/vesicles	65	DLS		[170]
	Purified	0.5	RT	8.5		Micelles	25	DLS		[170]
	Purified	0.5	RT	9.5		Micelles	25	DLS		[170]
	Purified	0.5	RT	10.5		Micelles	25	DLS		[170]
	99	0.02	25	6.8		Vesicles	100 * 85 **	DLS	0.73	[179]
	99	0.04	25	6.8		Vesicles	85 * 70 **	DLS	0.73	[179]
	99	0.1	25	6.8		Micelles/vesicles	85 * 50 **	DLS	0.73	[179]
	99	0.5	25	6.8		Micelles/vesicles	85 * 25 **	DLS	0.73	[179]
	99	1	25	6.8		Micelles/vesicles	80 * 30 **	DLS	0.73	[179]
	99	10	25	6.8		Micelles/vesicles	75 * 50 **	DLS	0.73	[179]
	99	20–100	25	6.8		Micelles/vesicles	2–4, 75–90 * 2–4 **	DLS	0.73	[179]

^§^ DLS (dynamic light scattering), SEM (scanning electron microscopy), POM (polarized optical microscopy), SANS (small-angle neutron scattering), TEM (transmission electron microscopy), SAXS (small-angle X-ray scattering). ^†^ Mono-rhamnolipids 98%, Rha-C_10_-C_10_ 72%. ^††^ Rha-C_10_-C_10_, Rha-C_10_-C_12_, and Rha-C_10_-C_12:1_. ^†††^ Rha-Rha-C_10_-C_10_ 50%, Rha-Rha-C_10_-C_12_ 29%. * Intensity-based particle size distributions. ** Number-based particle size distributions. ^a^ Inner radius. ^b^ Outer radius.

Small R1 aggregates, with radii on the order of a few nanometers, were detected and analyzed by different research groups [174,199] by means of DLS, fluorescence, and SANS.

In intensity-weighted DLS profiles of R1 solutions at pH 8 and concentrations ranging from 5 to 20 mM, Eismin et al. found three populations, *R_h_* = 2.4, 10, and 90 nm, with only the first population still visible in number-weighted profiles [174]. This result indicates that the most abundant species was the smallest one at all R1 concentrations investigated. The shape of these aggregates was first deduced by comparison between experimentally determined *R_h_* and the molecular length plus an arbitrary hydration shell as derived from molecular modeling [174]; a ratio of *R_h_* on the molecular length (*ee* parameter) higher than 1 was obtained, pointing toward an ellipsoidal micellar shape. Structural features of these objects were further investigated by means of fluorescence experiments with different probes. By means of fluorescence spectroscopy, the authors distinguished between aggregates formed below and above 7.5 mM; in the former case, aggregates were loosely packed allowing significant water penetration and they were classified as pre-micelles, whereas, in the latter case, water penetration into aggregates decreased, as they were much more closely packed, and these aggregates, characterized by aggregation numbers of about 26, were identified as “true” micelles [174]. The authors corroborated their hypothesis of pre-micellar aggregation by means of MD simulations, as detailed in Section 3.3, observing formation of an aggregate composed of seven monomers in systems containing 10 R1 molecules. Moreover, they found that hydrogen bonding interactions between rhamnose headgroups stabilized these kinds of aggregates, favoring pre-micellization [174]. The formation of aggregates of multiple monomers taken together by intermolecular interactions prior to the formation of full micelles has been postulated for multiple surfactant systems [200,201,202,203,204], but a significant difference with respect to the R1 case concerns the surfactant concentration at which it takes place; in other cases, for pre-micelles formed at concentrations below or very close to the *cmc*, in the R1 case, they should exist up to concentrations of about 50 times the *cmc*. Therefore, it is our opinion that some caution should be taken before identifying the loose aggregates experimentally observed in solution as pre-micelles.

Chen et al. characterized R1 aggregation at pH 9 by means of SANS in the 20–100 mM range, finding small micellar aggregates only at the lowest concentration, and lamellar arrangements starting from 30 mM concentration [199]. According to fitting of SANS data with a core–shell model, they described the micelles as elliptical (*ee* parameter 2.2), with an inner radius of about 1.4 nm and an outer radius of about 1.7 nm, an aggregation number of 47 monomers, and a surface charge of 5 [199]. This surface charge corresponds to a degree of micelle ionization of about 0.15, low compared to values of 0.2–0.3 generally encountered in ionic micelles of similar size [205], again arguing against a fully anionic nature of rhamnolipids even at pH 9. This finding is further supported by the relative insensitivity of the R1 micelle geometry to the addition of Ca^2+^ in solution, an index of the weak binding of cations to the predominantly nonionic rhamnolipid [199].

R2 forms small aggregates similarly to R1 at pH = 9, as clearly evidenced by SANS. With respect to R1, R2 micelles are found in a larger range of concentrations (20–100 mM), as an increase in concentration was found to determine an increase of the aggregation number, from 26 to 34 to 86 by moving from 20 to 50 to 100 mM, respectively, along with no significant morphological change [176]. Such small aggregates were identified as elliptical micelles, with an inner radius of about 1.2 nm, an outer radius of about 1.5 nm, and a surface charge of 2, indicating a degree of micelle ionization even lower than that found for R1 [176].

Above the rhamnolipid pKa, most researchers found R1 and R2 aggregates with larger dimensions than a few nanometers.

In the case of R1, the *R_h_* of aggregates is in the order of tens of nanometers (10–50 nm) with the most frequent value of about 25 nm, as determined by DLS [170,179,197]. These aggregates could be identified as micelles, but their dimensions suggest that they are elongated micelles. An increase in rhamnolipid concentration at fixed pH and room temperature determines a significant increase in dimensions that could be due to a transition from micellar to vesicular aggregates [170]; however, the actual concentration at which such a structural rearrangement occurs is not univocally defined. Wu et al., for example, only discussed the change in dimensions but did not attempt to define the nature of the objects they characterized with DLS [170]. Abbasi et al. observed the presence of aggregates with *R_h_* ~25 nm in the vicinity of the *cmc* (about 0.3 mM), and with *R_h_* ~100 nm at a concentration 10 times higher (3 mM). On the basis of the low turbidity and the close similarity of the polydispersity index of the two samples, they hypothesized that these aggregates were not large structures and were similar in both samples, concluding that, up to 3 mM R1 concentration, no lamellar arrangements (vesicles) are formed [71]. On the other hand, Chen et al., on the basis of SANS results, claimed that R1 forms small globular elliptical micelles with aggregation numbers of about 50 at about 20 mM concentration, while, at higher concentrations, it forms predominantly planar structures, unilamellar or bilamellar vesicles. Hence, they reported a micelle-to-vesicle transition occurring at significantly high concentrations, i.e., 50–100 mM [199]. Lastly, Eismin et al. observed larger aggregates in the compresence of small micelles at concentrations ranging from 0.1 to 100 mM and identified them as vesicles by means of fluorescence spectroscopy. However, in their case, the micelles were the most abundant among the different species at all the concentrations, and only the polydispersity increased with the R1 concentration [174].

R2 behaves very similarly to its analogous counterpart with just one rhamnose moiety in the headgroup; it forms both smaller aggregates (probably elongated micelles) with radii ranging between 15 and 35 nm, with the most frequent value found equal to 25 nm, and larger aggregates with radii ranging between 70 and about 300 nm. Conversion from one form of aggregates to the other occurs, as in the case of R1, upon increasing the concentration; however, R2 is reported to be able to arrange in micellar assemblies up to higher concentrations with respect to R1. At pH 6.8, a purified sample of this rhamnolipid was shown to form micelles of about 25 nm radius between 0.05 and 1 mM concentration [170], while a mixture of di-rhamnolipids with R2 representing the most abundant species at pH 7.4 was shown to form micelles of similar dimensions coexisting with larger aggregates, probably vesicles, between 0.125 and 1 mM [59]. By increasing the concentration beyond 1 mM at pH 7.4, only vesicles remained visible, in a few cases together with very large aggregates with radii larger than 500 nm, associated with elongated vesicles [59]. The transmission electron microscopy (TEM) characterization of R2 vesicles at concentrations higher than 2.5 mM indicated that they are formed by several lamellae, a finding supported by small-angle X-ray scattering (SAXS) data indicating the presence of lamellar multilayers at 20 mM concentration [59]. An opposite trend of morphological changes with concentration was described for R2 by İkizler et al. [179]; by means of DLS, they showed the formation of vesicles at very low concentrations (0.02–0.04 mM), as well as coexistence of micelles and vesicles in a wide range of concentrations from 0.1 to 100 mM, with very small aggregates (*R_h_* of a few nanometres) only present at the highest concentrations employed [179]. Such findings contrast most results obtained not only with rhamnolipids, but also with different glycolipids [206] and other surfactants [207,208,209,210], describing a micelle-to-vesicle transition induced by an increase in concentration.

Keeping the rhamnolipid concentration fixed at 0.5 mM, slightly higher than the *cmc*, and by increasing the temperature at pH = 6.8, Wu et al. observed a significant increase in aggregate dimensions for both R1 and R2, with the hydrodynamic radius changing from about 25 nm to more than 130 nm for R1 and to about 90 nm for R2, respectively [170]. This was interpretated as a micelle-to-vesicle transition occurring at about 45 °C [170]. Such temperature-induced transition has been observed for numerous surfactant systems [207,211,212]. The theoretical justification of this behavior rests on the fact that, at high temperature, the surfactant molecules are expected to be less hydrated and more hydrophobic, and a decrease in the head area [213,214] results in a larger value of the *cpp*. Therefore, a temperature increase promotes the formation of aggregates with lower curvature than micelles, such as vesicles.

The effect of pH was investigated at concentrations close to the *cmc* for both rhamnolipids. In both the cases, the dimension of the aggregates was found to decrease with increasing pH. For R1, below the pKa (pH 2.5, 3.5, 4.5), *R_h_* of more than 50 nm was determined, suggesting the presence of vesicles, whereas, above the pKa and up to neutral pH (7.5), aggregates with *R_h_* of about 35 nm were the main species present in solution and they were likely to be elongated micelles. Lastly, upon further increasing the pH, a gradual decrease in dimensions was observed. For R2, only vesicles were likely to be present below the pKa (pH 2.5, 3.5, 4.5), in contrast to coexisting vesicle and micelles for 5.5 ≤ pH ≤ 7.5, and only micelles for pH ≥ 8.5 [170]. This behavior was ascribed to the dissociation degree of the carboxylic group [215]. With the increase in pH, the number of ionized rhamnolipids increases; thus, electrostatic repulsion among the headgroups also increases, favoring the formation of aggregates with larger curvature (vesicle-to-micelle transition) and smaller dimensions.

Lastly, at pH > pKa, electrolytes affect the aggregation behavior of R1; the screening of the negative charges and the binding of ions to the polar head promotes the formation of larger aggregates with smaller curvature, such that addition of NaCl was found to cause a transition from micellar to lamellar structures [175], similarly to what happened upon increasing the rhamnolipid concentration and the temperature or upon decreasing the pH. No systematic analysis of the effect of electrolytes on R2 aggregation was reported, but a comparison between measurements performed at pH 9 in the absence and in the presence of calcium ions highlighted minimal structural changes of R2 micelles, confirming the very slight ionic nature of this rhamnolipid [199].

The effect of electrolytes was amply discussed by Helvacy and Ozdemir, who reported that R1 and R2 formed spherical micelles in the absence of electrolytes, and that, upon increasing the NaCl concentration, a micelle-to-vesicle and then a vesicle-to-rod-like micelle transition occurred [172]. However, despite their results often being cited in the literature, particularly as an indication that rhamnolipids may form spherical micelles, they should be reconsidered as several questionable assumptions were made to support their conclusions. First, they calculated a *cpp* = 0.24 for R1 and 0.27 for R2 in the absence of electrolytes and related these values to a preferential aggregation into spherical micelles, but derived the mean molecular area *a_e_* for *cpp* calculation from surface tension data, i.e., from the intercept of the tangent line drawn at the compaction end of the surface pressure–molecular area (π–A) isotherm evaluated from surface tension data [172]. In this respect, as discussed in Section 2.2.3, they likely overestimated the extent of rhamnolipid dissociation and, consequently, the area per molecule at the interface, resulting in abnormally large values of *a_e_*, from which unusual small *cpp* values are obtained. Indeed, most authors agree on the fact that rhamnolipid only forms ellipsoidal/elongated micelles, in addition to lamellar aggregates, incompatible with *cpp* <1/3. Furthermore, as further support for the spherical nature of rhamnolipid micelles, Helvacy and Ozdemir brought into play the presence of small Maltese crosses in POM images [172], whereas they were only indicative of the presence of anisotropic lamellar aggregates [216,217,218,219].

Overall, R1 and R2 aggregation behavior is similar, despite the different size of the headgroups; a tendency toward the formation of aggregates with low curvature, likely multilamellar vesicles, is envisaged, and, in particular, aggregation into lamellar arrangements is promoted by high temperatures and concentrations, low pH, and, at pH > pKa, by an increase in ionic strength. The scarce influence of the number of rhamnose moieties on the aggregation behavior of rhamnolipids agrees well with the very close *cpp* values determined for R1 and R2, notwithstanding all the precautions required for calculation of this parameter; for R1, *cpp* values of 0.5–0.6 are mostly reported, whereas, for R2, the mean value is 0.7. These values predict the actual kind of aggregates observed, as surfactants with ⅓ < *cpp* < ½ are known to form cylinders and those with *cpp* > ½ are known to form vesicles [193]; indeed, both R1 and R2 form either elongated micelles or ellipsoidal, cylindrical, or lamellar structures such as vesicles.

Overall, R2 micelles are stable in a larger range of rhamnolipid concentrations than R1, but both rhamnolipids undergo a micelle-to-vesicle transition upon increasing their concentration. However, strong discrepancies exist in the actual kind of aggregates formed at a given concentration. In this respect, it should always be taken in mind that rhamnolipid samples rarely present the same purity grade, and purified rhamnolipids are often still a mixture of congeners, with different compositions of the apolar tail, and this may be the reason for the significant differences encountered.

### 3.2. Synthetic Rhamnolipids

Unfortunately the structural characterization of aggregates formed by highly pure synthetic rhamnolipids has up to now been very limited, and only few hints on the effects of tail symmetry and stereochemistry can be deduced on the basis of studies by the research group of Pemberton [147,148]. The authors probed the aggregation features of the four diastereomers of Rha-C_10_-C_10_ by means of fluorescence spectroscopy using pyrene as a probe [147]. They found a practically indistinguishable behavior for the four molecules, very close to that of the natural mono-rhamnolipid, indicating that stereochemistry has a negligible effect on the aggregation of rhamnolipids. Specifically, Palos Pacheco et al. observed the full incorporation of the probe within what they supposed to be well-formed aggregates only at concentrations well above the *cmc* (>5 mM), and they interpreted their findings on the basis of the formation of pre-micelles, similarly to what was described in the case of the mono-rhamnolipids purified by *Pseudomonas aeruginosa* ATCC 9027 (as described in Section 3.1) [174].

Much more interesting is the effect of modifications of the hydrophobic portion [148]. Mono-rhamnolipids with one tail of 14 carbon atoms and a second tail with variable length from C_6_ to C_14_ all form three kinds of coexisting aggregates: small micelles with *R_h_* of a few nanometers, and larger aggregates with *R_h_* = 5–20 nm and *R_h_* = 50–100 nm. The authors suggested that larger aggregates may be unilamellar and multilamellar vesicles, respectively [148]; however, on the basis of the results discussed above, the population with *R_h_* on the order of tens of nanometers may also be due to cylindrical micelles. The smallest aggregates are the most abundant ones for all congeners in the concentration range 0.1–30 mM. However, as the length of the second tail increases, larger aggregates become more abundant, with significant polydispersity of solutions of Rha-C_14_-C_10_, Rha-C_14_-C_12_, and Rha-C_14_-C_14_ [148]. Interestingly, the polarity experienced by pyrene appears higher in vesicular aggregates formed by more symmetric congeners than in micellar ones formed by more asymmetric congeners, in contrast with findings obtained for different surfactants [220]. Such an unexpected result was justified on the basis of a strong tail–tail interaction in symmetric rhamnolipids, causing changes in the headgroup conformation and pushing the pyrene outward toward the more polar headgroup region [148]. In this respect, it would be legitimate to wonder how other factors, particularly the rhamnolipid concentration, may affect the head conformation, and whether the pre-micellar aggregates identified by the Pemberton group on the basis of fluorescence results [174] were not simply micellar aggregates differing in terms of head conformation.

### 3.3. Computational Results

The aggregation behavior of rhamnolipids has also been investigated by means of molecular dynamics simulations [174,187,189,221]. However, it is worth recalling some limitations of computational approaches for the prediction of aggregates formed by these surfactants. The timescale for the formation of micellar and vesicular aggregates is longer than that usually analyzed by MD [189]; therefore, a simulated annealing [222] approach is always employed. The time evolution of arbitrary states is analyzed upon heating, equilibration at high temperature, and slow cooling down to room temperature [174,189,221]; however, in this way some structures can escape investigation, and others may not reflect the actual aggregates present in solution. Another difficulty arises from the constant evolution of aggregates, particularly association of small micelles into larger aggregates (cylindrical or wormlike micelles and vesicles) [189]. Nonetheless computational results complement the experimental data and provide some precious hints on the self-aggregation of rhamnolipids in water.

The aggregation behavior of both the anionic [174] and the nonionic [221] forms of Rha-C_10_-C_10_ was studied by MD simulations. In the former case, the authors started from systems containing a different number of rhamnolipid molecules, i.e., from 10 to 100 molecules. As briefly mentioned in Section 3.1, very small aggregates identified as pre-micelles and composed of 7 R1 molecules were observed in the system containing only 10 molecules. However, in this respect, the very limited dimension of the starting state is likely to strongly affect the results, and some caution when bringing this finding to support the pre-micellization of R1 should be adopted. In the largest system, containing 100 molecules, small aggregates of ∼25 monomers are observed, as well as merging of aggregates with N_agg_ < 25 into larger structures. On this basis, the authors suggested that an aggregation number of ∼25 is the most probable for R1 anionic micelles. Moreover, MD simulations showed that these micelles are almost spherical at low aggregation numbers (about 25 monomers/micelle) and gradually grow along one direction, becoming increasingly elliptical up to tubular upon increasing the R1 concentration and the consequent aggregation number [174].

Furthermore, in the case of the nonionic form of R1, very small aggregates (N_agg_ = 5–10) are observed in the smallest systems, but aggregates with N_agg_ = 8 and 16 are also found in systems composed of 50 and 60 molecules, respectively. However, the hydrophobic core of these aggregates is significantly exposed to the surrounding solvent and too small to stabilize them; therefore, they cannot be considered as stable micelles. The observation of aggregates with an aggregation number close to ∼40 in most simulations suggests that this may be the stable N_agg_ of nonionic R1. In support of this hypothesis, the authors found that larger aggregates, formed by 80 or 100 molecules, do not present a hydrophobic core enclosed by hydrophilic moieties, as expected for micelles, not even when simulated for longer times, unlike what has been found for anionic R1, which is able to form elongated, tubular aggregates with high aggregation numbers.

The dimension of micelles formed by nonionic R1 is close to experimental data obtained by means of SANS for anionic R1 (the average radius is about 2 nm), but the highly dynamic nature of these aggregates does not allow the exact morphology (spherical, ellipsoidal, and cylindrical) to be determined. Nonetheless, for N_agg_ = 40 aggregates, the eccentricity distributions provide an indication that the micelles may be ellipsoidal [221].

Interestingly, the different aggregation behavior of anionic and nonionic forms of R1 could be related to their different ability to form H-bonds between neighboring headgroups. While the anionic headgroups are involved in H-bond networks, stabilizing aggregates with N_agg_ > 40 [174], the nonionic headgroups are not; therefore, the stability of nonionic aggregates is only due to the hydrophobic interactions between the tails, and their limited length (only seven carbon atoms) hampers the formation of aggregates larger than about 40 molecules [221].

In the case of the nonionic mono-rhamnolipids, Munusamy et al. also investigated using MD simulations the stability of large aggregates formed by hundreds of molecules, namely, a torus-like aggregate and a unilamellar vesicle, finding that both are highly stabilized by the hydrophobic bilayer, and that the presence of structured water molecules within the hollow core of vesicles further stabilizes vesicular aggregates [221].

From a morphological viewpoint, the preference of anionic R1 to form micellar aggregates in addition to large lamellar vesicles, and of nonionic R1 to form lamellar vesicles over small micellar aggregates can also be related to the lack of the hydrogen bonding interactions between the nonionic monomers in small micelles, driving the formation of larger lamellar vesicles stabilized by a strong hydrophobic interaction within the bilayer arrangement of the tails [187].

A similar investigation was also performed for the di-rhamnolipid Rha-Rha-C_10_-C_10_ in its nonionic form, as well as for Rha-Rha-C_14_-C_14_ and Rha-Rha-C_18_-C_18_, in order to test the effects of tail length [189]. MD simulations highlight that R2 has a tendency to form aggregates smaller than those formed by R1, with N_agg_ = 22 and an almost spherical shape. These aggregates present a good separation between hydrophobic and hydrophilic components and can, therefore, be considered full-fledged micelles. On the contrary, no such separation is detected for aggregates with N_agg_ higher or lower than 22, which are, therefore, merely interacting monomers [189]. The authors related this behavior to an imbalance between the hydrophilic and hydrophobic components of R2 due to the presence of the additional rhamnose moiety [189]; on one hand, the larger headgroup requires greater solvation than the mono-rhamnolipid and this should drive the mean aggregation number down [223], while, on the other hand, it hampers stabilization of larger aggregates. In support of the latter point, the increase in size of the hydrophobic region in Rha-Rha-C_14_-C_14_ and Rha-Rha-C_18_-C_18_ results in the formation of micellar aggregates with N_agg_ ~30 that grow with the concentration evolving from spherical to cylindrical micelles.

Overall, the self-aggregation behavior in bulk solution of mono- and di-rhamnolipids is quite similar; both show a tendency toward the formation of elongated micelles and aggregates with low curvature such as vesicles. Moreover, changes in solution conditions, such as pH, temperature, concentration, or ionic strength, induce the transition from one kind of aggregate to the other. Interestingly experimental and computation data highlight the significant influence played by the length and symmetry of the hydrophobic region of the molecules, while computational results also provide a justification of the different behavior of anionic and nonionic forms of R1, depending on their different ability to form hydrogen bonds between the headgroups.

## 4. Open Issues

The characterization of rhamnolipid behavior in aqueous solutions and at the air/water interface also includes a few examples focusing on surfactant mixtures composed of rhamnolipids and other surfactants, either anionic or nonionic ones, namely, sodium dodecylbenzenesulfonate [176,199], sodium dodecylsulfate [224], sodium laurylethersulfate [225], lactonic sophorolipid [226], the aliphatic alcohol ethoxylate surfactant tergitol 15-S-7 [227], Triton X-100 [228], and Triton X-165 [229]. These studies highlight deviations from the ideal mixing pointing toward synergistic interactions between rhamnolipids and sophorolipid [226], sodium dodecylsulfate [224], and both Triton surfactants [228,229]. On the other hand, antagonistic interactions take place between rhamnolipids and sodium laurylethersulfate [225], while the scenario is more complex in the case of dodecylbenzenesulfonate, with an ideal behavior for mixtures containing R1 and deviations from ideality for mixture containing R2 or both R1 and R2 [176]. Such limited results do not allow a full understanding of the interactions established between rhamnolipids and other surfactants. In this respect, the rhamnolipid behavior in mixtures with other surfactants, which may be nonionic, anionic, or cationic, deserves further investigation in the future not only for scientific, but also for applicative reasons. On one hand, synergism of surfactant mixtures is amply exploited for enhancement of formulation performances [230]; on the other hand, the possibility to form catanionic mixtures based on rhamnolipids can be investigated for the delivery of several actives [220].

What is mostly unexplored is rhamnolipid self-aggregation in concentrated mixtures. Formation of lyotropic liquid crystalline (LLC) phases has been proposed in some papers on the basis of quite weak experimental evidence [86,171], whose reliability was later questioned [4], as well as on the basis of computational simulations [231]. For conventional surfactants, the formation of LLC phases and the transition between them has been rationalized using the theory of colloid stability [232], which considers various contributions, including electrostatic, electrolyte adsorption [233], curvature and bending of the aggregate surface [234], and van der Waals energies [235]. However, to date, no similar approach has been used for biosurfactants in general and rhamnolipids in particular. A poor scientific understanding of concentrated rhamnolipid systems severely impacts their possible industrial applications, as ecological concerns arising from CO_2_ emissions and plastic packaging disposal, connected to the large volumes of surfactant formulations transported and marketed worldwide, is driving formulation scientists and technologists toward the design of water-poor products [236].

Lastly, the investigation of salt effects on rhamnolipid self-aggregation has been very limited, as discussed in the previous section; no effort has been devoted to the analysis of salts other than sodium chloride, and salt addition has been considered only with respect to an increase in ionic strength. This represents a severe weakness of these studies, because ion effects strongly depend on the nature of the ions, which can be classified according to Hofmeister or reversal Hofmeister series [237,238,239,240,241]. Some crucial issues concern the fact that ions affect all possible interactions in a considered system [242], and that many phenomena involve the action of both ions of an electrolyte; thus, one needs to consider both of them. For all these reasons, and taking into account the structural complexity of rhamnolipids and the inherent flexibility of their headgroup, a systematic study on ion-specific effects with respect to bulk and surface physicochemical properties of rhamnolipids should be undertaken in the future, and the results should be rationalized in the context of currently employed theoretical models [243,244,245,246]. Achieving a good understanding of ion-specific effects on rhamnolipid behavior is a necessary step toward the replacement of conventional surfactants with rhamnolipids in technological applications, given how deeply ions affect formulation features.

## 5. Conclusions and Future Perspectives

Rhamnolipids are a class of biosurfactants with great potential for the replacement of synthetic surfactants in a wide range of applications. Impressive scientific and technological progress has been achieved, and effort is still devoted toward optimization of their production processes to decrease production costs making them competitive with petrochemical-derived surfactants. At the same time, their physicochemical properties, particularly their self-aggregation in aqueous solutions, have been analyzed as a function of rhamnolipid structure and environmental conditions. However, despite a consistent amount of data on rhamnolipid behavior, a conclusive structure–function relationship is far from being defined. The comprehensive review of rhamnolipid physicochemical properties we presented here allows us to move a step forward in the definition of a structure–function relationship, particularly by disproving rooted beliefs.

On the basis of molecular structure, one would expect a different behavior for mono- and di-rhamnolipids; indeed, several studies assumed a strong effect of the bulky headgroup of di-rhamnolipids to exist. On the contrary, we highlight a negligible effect of the second rhamnose moiety, for what concerns self-aggregation at the air/water interface and in bulk solution; mono- and di-rhamnolipids are characterized by very similar *cmc*, *γ_min_*, and *A_min_* values and form similar kinds of aggregates in aqueous solution, which undergo morphological transitions in response to changes in rhamnolipid concentration, temperature, pH, and ionic strength.

The presence of a carboxylic group is supposed to confer to rhamnolipids a strong pH dependence; indeed, the solution pH affects the self-aggregation of both mono- and di-rhamnolipids, with *cmc* values decreasing and the aggregate nature changing from micellar to vesicular with a decrease in pH. However, a strict comparison of experimental data shows that the effects of pH on the minimum surface tension and maximum surface concentration are almost negligible. In this respect, it is worth stressing that complementary experimental investigations, e.g., by means of neutron reflectivity or polarization modulated-infrared reflection absorption spectroscopy, as well as computational results from molecular dynamics simulations, indicate that determination of maximum surface concentration and minimum molecular area at the interface by fitting of tensiometry data are strongly biased by the choice of the prefactor used in the Gibbs equation and can give wrong results. At pH above the pKa, the effect of ionic strength on self-aggregation is detectable but much less significant than that found for conventional ionic surfactants. Overall, rhamnolipids present a very slight anionic character at pH above the pKa, and this is more so true for di-rhamnolipids than for mono-rhamnolipids.

**Figure 3:**
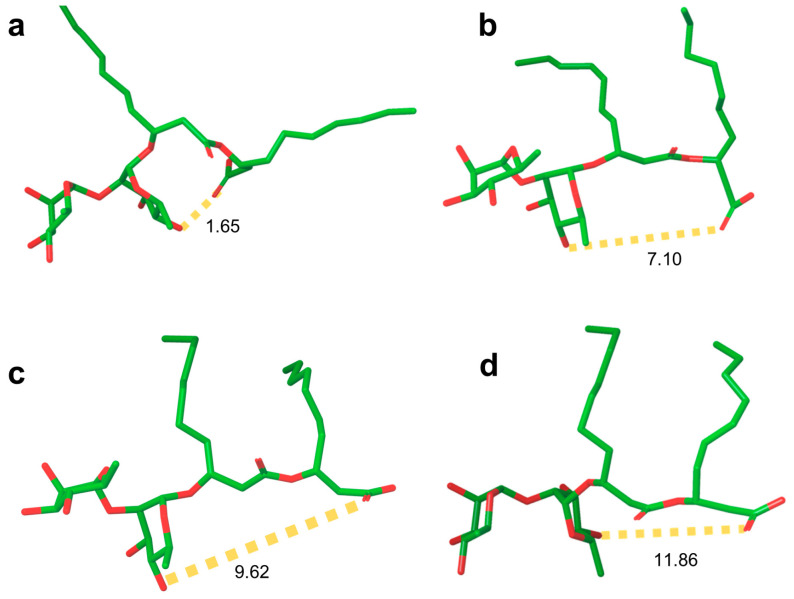
Representative spatial arrangements of di-rhamnolipid molecule (Rha-Rha-C_10_-C_10_) obtained as result of molecular dynamic simulation highlighting headgroup conformational flexibility. The three structures differ for the potential energy value: (**a**) 39.019 kJ/mol (medium energy) (corresponding to the closed conformation described by Luft et al. [189]), (**b**) 38.892 kJ/mol (local minimum energy) (corresponding to the open conformation described by Luft et al. [189]), (**c**) 37.967 kJ/mol (global minimum energy), (**d**) 42.255 kJ/mol (highest energy). The H-bond distance between the carboxyl group and the -OH at position C4 of the first rhamnose is explicitly shown. The structures were built using the Built facility in Maestro program in the Schrödinger suite (Maestro, Schrödinger, LLC, New York, NY, USA). MD parameters: AMBER* force field, water as solvent, at 298 K, 100 ns simulation time, monitoring 10,000 structures.

Both the close similarity between mono-and di-rhamnolipids and the poor ionic character of these biosurfactants above their pKa can be related to the high conformational flexibility of the headgroup, as shown by MD simulations. Indeed, the adoption of a folded structure for di-rhamnolipids results in a significant reduction in the headgroup volume, making it not so dissimilar from that of mono-rhamnolipids, despite the presence of the additional rhamnose moiety. Furthermore, the headgroup of both rhamnolipids can adopt a closed conformation with the formation of a sort of pocket in which the anionic charge can be buried along with its counterion, making rhamnolipids behave as nonionic surfactants even when they bear a net negative charge (Figure 3).

While modifications of the rhamnolipid headgroup structural features have scarce effects on determining their self-aggregation properties, the molecular structure of the hydrophobic region of these biosurfactants seems to play a crucial role, with significant effects of alkyl chain length, as well as of the asymmetry between tails. What is intriguing in the case of rhamnolipids is that, thanks to the blurred distinction between hydrophilic and hydrophobic portions of the molecule and the conformational flexibility of the headgroup, a modification of the tails can also induce changes in the headgroup, thus giving unexpected and unpredictable results in terms of intermolecular interactions and consequent self-aggregation. This aspect is very difficult to investigate given the inherent heterogeneity of rhamnolipids with regard to the composition of the hydrophobic tails.

Aiming at defining structure–function relationships and elaborating theoretical models able to rationalize and predict the behavior of rhamnolipids, it would be highly desirable in the future to have the possibility to investigate pure molecules obtained by means of synthetic or biotechnological approaches or through purification of natural mixtures, as the heterogeneity of rhamnolipid solutions with the coexistence of several congeners often hampers a straightforward interpretation of results. Moreover, a thorough characterization of their aggregation behavior in solution, with a strict definition of the kinds of aggregates formed as a function of temperature and, in particular, rhamnolipid concentration, appears necessary for the definition of a complete phase diagram.

We believe that a deep understanding of the molecular determinants of rhamnolipid physicochemical properties would be beneficial for a large scientific community, by providing a basis for the rational design of rhamnolipid-based formulations and expanding their use in different applications, particularly in refined applications such as medical and pharmaceutical ones.

## Figures and Tables

**Table 1:** Rhamnolipid congeners produced by *Pseudomonas aeruginosa* using either glucose or soy as a carbon source [145]. The length of the tails is indicated by the first subscript number, while the second subscript number indicates the degree of unsaturation. For example, C_12:2_ indicates a tail 12 carbon atoms long with two degrees of unsaturation.

Mono-Rhamnolipid Mono-Lipidic								
**Congener**	**Molar Mass** **(g·mol^−1^)**	**Relative Abundance (wt.%)** **Glucose Substrate**	**Relative Abundance (wt.%)** **Soy Substrate**	**R1**	**n1**	**n2**	**R2**	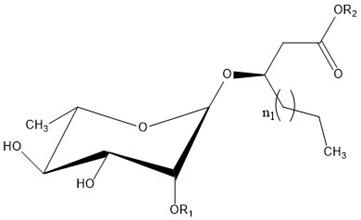
Rha-C_6_	277	2	-	-	-	-	-	
Rha-C_8_	305	15	4	H	1	-	H	
Rha-C_8:2_	302	-	*	H	1(−4H)	-	H	
Rha-C_10_	333	7	6	H	3	-	H	
Rha-C_12:2_	358	-	*	H	5(−4H)	-	H	
Rha-C_12_	362	-	7	H	5		H	
Rha-C_14:2_	386	2	-	H	7(−4H)	-	H	
Rha-C_16:1_	415	15	4	-	-	-	-	
**Mono-Rhamnolipid Di-Lipidic**								
**Congener**	**Molar Mass** **(g·mol^−1^)**	**Relative Abundance (wt.%)** **Glucose Substrate**	**Relative Abundance (wt.%)** **Soy Substrate**					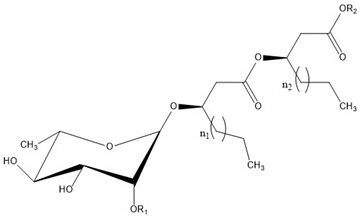
Rha-C_6_-C_8_	419	^-^	2	-	-	-	-	
Rha-C_8_-C_8_	447	5	2	H	1	3(−2H)	H	
Rha-C_8_-C_8:1_	445	-	3	-	-	-	-	
Rha-C_8_-C_10_	475	18	-	H	1	3	H	
Rha-C_8_-C_10:1_	473	-	18					
Rha-C_10_-C_10_	503	7	14	H	3	3	H	
Rha-C_10_-C_12_	531	4	5	H	3	5	H	
Rha-C_12_-C_12:1_	557	4	-	H	5	5(−2H)	H	
**Di-Rhamnolipid Mono-Lipidic**								
**Congener**	**Molar Mass** **(g·mol^−1^)**	**Relative Abundance (wt.%)** **Glucose Substrate**	**Relative Abundance (wt.%)** **Soy Substrate**					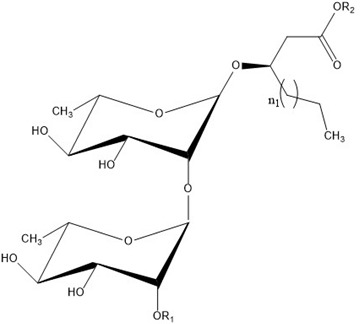
Rha-Rha-C_10_	479	13	2					
**Di-Rhamnolipid Di-Lipidic**								
**Congener**	**Molar Mass** **(g·mol^−1^)**	**Relative Abundance (wt.%)** **Glucose Substrate**	**Relative Abundance (wt.%)** **Soy Substrate**					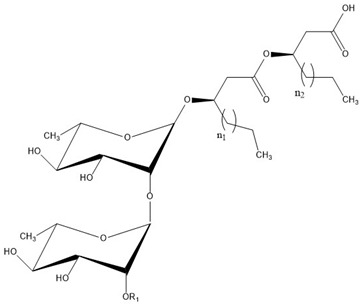
Rha-Rha-C_10_-C_10_	649	13	11	H	3	3	H	
Rha-Rha-C_10_-C_12_	677	-	4	H	3	5	H	
Rha-Rha-C_12_-C_12_	705	1	-	H	5	5	H	
Rha-Rha-C_6_-C_12_	621	-	7					
Rha-Rha-C_6_-C_14_	649	-	2					
Rha-Rha-C_8_-C_10_	621	5	4	H	1	3	H	
Rha-Rha-C_8_-C_12_	649	3	6					
Rha-Rha-C_8_-C_12:1_	647	3	5	H	1	5(−2H)	H	

* Relative abundance not reported in Abalos et al. [99].

**Table 2:** The *cmc* and surface activity of microbial rhamnolipid characterized as crude extracts.

Organism	Carbon Source	pH	Conditions	*cmc* (mg/L)	*γ_min_*	Predominant Rhamnolipid Homologues	Ref
*Pseudomonas aeruginosa* L2-1	Mineral salts medium (MSM) + 2% (*w*/*v*) soybean oil			30	30	Rha-Rha-C_10_-C_10_	[76]
*Pseudomonas aeruginosa* #112	Corn steep liquor (10%, *v*/*v*) + sugarcane molasses (10%, *w*/*v*)		Flask	50	31.4	Rha-C_10_-C_10_; Rha-Rha-C_10_-C_10_; Rha-C_10_	[110]
*Pseudomonas aeruginosa* #112	Corn steep liquor (10%, *v*/*v*) + sugarcane molasses (10%, *w*/*v*)		Reactor	30	29.0	Rha-C_10_-C_10_; Rha-Rha-C_10_-C_10_; Rha-C_10_	[110]
*Pseudomonas aeruginosa* #112	Corn steep liquor (10%, *v*/*v*) + sugarcane molasses (10%, *w*/*v*) +5% oil mill wastewater			38	31.3	Rha-C_10_-C_10_; Rha-Rha-C_10_-C_10_; Rha-C_10_	[110]
*Pseudomonas aeruginosa* #112	Corn steep liquor (10%, *v*/*v*) + sugarcane molasses (10%, *w*/*v*) +10% oil mill wastewater			36	31.4	Rha-C_10_-C_10_; Rha-Rha-C_10_-C_10_; Rha-C_10_	[110]
*Pseudomonas aeruginosa* #112	Corn steep liquor (10%, *v*/*v*) + sugarcane molasses (10%, *w*/*v*) +15% oil mill wastewater			34	31.3	Rha-C_10_-C_10_; Rha-Rha-C_10_-C_10_; Rha-C_10_	[110]
*Pseudomonas aeruginosa* #112	Corn steep liquor (10%, *v*/*v*) + sugarcane molasses (10%, *w*/*v*) +20% oil mill wastewater			15	31.0	Rha-C_10_-C_10_; Rha-Rha-C_10_-C_10_; Rha-C_10_	[110]
*Pseudomonas aeruginosa* #112	Corn steep liquor (10%, *v*/*v*) + sugarcane molasses (10%, *w*/*v*) +25% oil mill wastewater		Flask	14	31.0	Rha-C_10_-C_10_; Rha-Rha-C_10_-C_10_; Rha-C_10_	[110]
*Pseudomonas aeruginosa* #112	Corn steep liquor (10%, *v*/*v*) + sugarcane molasses (10%, *w*/*v*) +25% oil mill wastewater		Reactor	13	29.2	Rha-Rha-C_10_-C_10_	[110]
*Pseudomonas aeruginosa* NCIM 5514	Bushnell-Hass (BH) medium (pH 7.2) supplemented with 1% (*w*/*v*) glucose		T = 4 °C		36.85 ^a^ 38.20 ^b^ 39.43 ^c^	Rha-C_10_-C_14:1_; Rha-C_8_-C_10_	[67]
*Pseudomonas aeruginosa* NCIM 5514	Bushnell-Hass (BH) medium (pH 7.2) supplemented with 1% (*w*/*v*) glucose		T = 30 °C		34.23 ^a^ 37.98 ^b^ 39.78 ^c^	Rha-C_10_-C_14:1_; Rha-C_8_-C_10_	[67]
*Pseudomonas aeruginosa* NCIM 5514	Bushnell-Hass (BH) medium (pH 7.2) supplemented with 1% (*w*/*v*) glucose		T = 37 °C		34.05 ^a^ 38.76 ^b^ 40.76 ^c^	Rha-C_10_-C_14:1_; Rha-C_8_-C_10_	[67]
*Pseudomonas aeruginosa* NCIM 5514	Bushnell-Hass (BH) medium (pH 7.2) supplemented with 1% (*w*/*v*) glucose		T = 40 °C		33.57 ^a^ 37.12 ^b^ 39.67 ^c^	Rha-C_10_-C_14:1_; Rha-C_8_-C_10_	[67]
*Pseudomonas aeruginosa* NCIM 5514	Bushnell-Hass (BH) medium (pH 7.2) supplemented with 1% (*w*/*v*) glucose		T = 50 °C		33.21 ^a^ 35.87 ^b^ 40.19 ^c^	Rha-C_10_-C_14:1_; Rha-C_8_-C_10_	[67]
*Pseudomonas aeruginosa* NCIM 5514	Bushnell-Hass (BH) medium (pH 7.2) supplemented with 1% (*w*/*v*) glucose		T = 60 °C		32.96 ^a^ 34.75 ^b^ 41.06 ^c^	Rha-C_10_-C_14:1_; Rha-C_8_-C_10_	[67]
*Pseudomonas aeruginosa* NCIM 5514	Bushnell-Hass (BH) medium (pH 7.2) supplemented with 1% (*w*/*v*) glucose		T = 70 °C		31.82 ^a^ 34.76 ^b^ 37.23 ^c^	Rha-C_10_-C_14:1_; Rha-C_8_-C_10_	[67]
*Pseudomonas aeruginosa* NCIM 5514	Bushnell-Hass (BH) medium (pH 7.2) supplemented with 1% (*w*/*v*) glucose		T = 80 °C		32.53 ^a^ 35.80 ^b^ 42.30 ^c^	Rha-C_10_-C_14:1_; Rha-C_8_-C_10_	[67]
*Pseudomonas aeruginosa* NCIM 5514	Bushnell-Hass (BH) medium (pH 7.2) supplemented with 1% (*w*/*v*) glucose		T = 90 °C		32.90 ^a^ 36.29 ^b^ 43.79 ^c^	Rha-C_10_-C_14:1_; Rha-C_8_-C_10_	[67]
*Pseudomonas aeruginosa* NCIM 5514	Bushnell-Hass (BH) medium (pH 7.2) supplemented with 1% (*w*/*v*) glucose		T = 100 °C		33.65 ^a^ 36.87 ^b^ 44.97 ^c^	Rha-C_10_-C_14:1_; Rha-C_8_-C_10_	[67]
*Pseudomonas aeruginosa* NCIM 5514	Bushnell-Hass (BH) medium (pH 7.2) supplemented with 1% (*w*/*v*) glucose	2.0			32.48	Rha-C_10_-C_14:1_; Rha-C_8_-C_10_	[67]
*Pseudomonas aeruginosa* NCIM 5514	Bushnell-Hass (BH) medium (pH 7.2) supplemented with 1% (*w*/*v*) glucose	4.0			34.17	Rha-C_10_-C_14:1_; Rha-C_8_-C_10_	[67]
*Pseudomonas aeruginosa* NCIM 5514	Bushnell-Hass (BH) medium (pH 7.2) supplemented with 1% (*w*/*v*) glucose	6.0			34.83	Rha-C_10_-C_14:1_; Rha-C_8_-C_10_	[67]
*Pseudomonas aeruginosa* NCIM 5514	Bushnell-Hass (BH) medium (pH 7.2) supplemented with 1% (*w*/*v*) glucose	7.2			32.89	Rha-C_10_-C_14:1_; Rha-C_8_-C_10_	[67]
*Pseudomonas aeruginosa* NCIM 5514	Bushnell-Hass (BH) medium (pH 7.2) supplemented with 1% (*w*/*v*) glucose	8.0			33.41	Rha-C_10_-C_14:1_; Rha-C_8_-C_10_	[67]
*Pseudomonas aeruginosa* NCIM 5514	Bushnell-Hass (BH) medium (pH 7.2) supplemented with 1% (*w*/*v*) glucose	10.0			36.52	Rha-C_10_-C_14:1_; Rha-C_8_-C_10_	[67]
*Pseudomonas aeruginosa* NCIM 5514	Bushnell-Hass (BH) medium (pH 7.2) supplemented with 1% (*w*/*v*) glucose	12.0			39.43	Rha-C_10_-C_14:1_; Rha-C_8_-C_10_	[67]
*Pseudomonas aeruginosa* NCIM 5514	Bushnell-Hass (BH) medium (pH 7.2) supplemented with 1% (*w*/*v*) glucose		NaCl 0% *w*/*v*		33.56	Rha-C_10_-C_14:1_; Rha-C_8_-C_10_	[67]
*Pseudomonas aeruginosa* NCIM 5514	Bushnell-Hass (BH) medium (pH 7.2) supplemented with 1% (*w*/*v*) glucose		NaCl 0.5% *w*/*v*		33.12	Rha-C_10_-C_14:1_; Rha-C_8_-C_10_	[67]
*Pseudomonas aeruginosa* NCIM 5514	Bushnell-Hass (BH) medium (pH 7.2) supplemented with 1% (*w*/*v*) glucose		NaCl 1% *w*/*v*		33.27	Rha-C_10_-C_14:1_; Rha-C_8_-C_10_	[67]
*Pseudomonas aeruginosa* NCIM 5514	Bushnell-Hass (BH) medium (pH 7.2) supplemented with 1% (*w*/*v*) glucose		NaCl 1.5% *w*/*v*		32.99	Rha-C_10_-C_14:1_; Rha-C_8_-C_10_	[67]
*Pseudomonas aeruginosa* NCIM 5514	Bushnell-Hass (BH) medium (pH 7.2) supplemented with 1% (*w*/*v*) glucose		NaCl 2% *w*/*v*		32.96	Rha-C_10_-C_14:1_; Rha-C_8_-C_10_	[67]
*Pseudomonas aeruginosa* NCIM 5514	Bushnell-Hass (BH) medium (pH 7.2) supplemented with 1% (*w*/*v*) glucose		NaCl 2.5% *w*/*v*		33.00	Rha-C_10_-C_14:1_; Rha-C_8_-C_10_	[67]
*Pseudomonas aeruginosa* NCIM 5514	Bushnell-Hass (BH) medium (pH 7.2) supplemented with 1% (*w*/*v*) glucose		NaCl 3% *w*/*v*		32.85	Rha-C_10_-C_14:1_; Rha-C_8_-C_10_	[67]
*Pseudomonas aeruginosa* NCIM 5514	Bushnell-Hass (BH) medium (pH 7.2) supplemented with 1% (*w*/*v*) glucose		NaCl 3.5% *w*/*v*		32.53	Rha-C_10_-C_14:1_; Rha-C_8_-C_10_	[67]
*Pseudomonas aeruginosa* NCIM 5514	Bushnell-Hass (BH) medium (pH 7.2) supplemented with 1% (*w*/*v*) glucose		NaCl 4% *w*/*v*		30.47	Rha-C_10_-C_14:1_; Rha-C_8_-C_10_	[67]
*Pseudomonas aeruginosa* NCIM 5514	Bushnell-Hass (BH) medium (pH 7.2) supplemented with 1% (*w*/*v*) glucose		NaCl 4.5% *w*/*v*		30.68	Rha-C_10_-C_14:1_; Rha-C_8_-C_10_	[67]
*Pseudomonas aeruginosa* NCIM 5514	Bushnell-Hass (BH) medium (pH 7.2) supplemented with 1% (*w*/*v*) glucose		NaCl 5% *w*/*v*		29.71	Rha-C_10_-C_14:1_; Rha-C_8_-C_10_	[67]
*Pseudomonas aeruginosa* NCIM 5514	Bushnell-Hass (BH) medium (pH 7.2) supplemented with 1% (*w*/*v*) glucose		NaCl 6% *w*/*v*		29.84	Rha-C_10_-C_14:1_; Rha-C_8_-C_10_	[67]
*Pseudomonas aeruginosa* NCIM 5514	Bushnell-Hass (BH) medium (pH 7.2) supplemented with 1% (*w*/*v*) glucose		NaCl 8% *w*/*v*		30.95	Rha-C_10_-C_14:1_; Rha-C_8_-C_10_	[67]
*Pseudomonas aeruginosa* NCIM 5514	Bushnell-Hass (BH) medium (pH 7.2) supplemented with 1% (*w*/*v*) glucose		NaCl 10% *w*/*v*		31.07	Mixture Rha-C10-C14:1 and Rha-C8-C1	[67]
*Pseudomonas aeruginosa* NCIM 5514	Bushnell-Hass (BH) medium (pH 7.2) supplemented with 1% (*w*/*v*) glucose		NaCl 12% *w*/*v*		33.00	Rha-C_10_-C_14:1_; Rha-C_8_-C_10_	[67]
*Pseudomonas aeruginosa* NCIM 5514	Bushnell-Hass (BH) medium (pH 7.2) supplemented with 1% (*w*/*v*) glucose		NaCl 14% *w*/*v*		33.50	Rha-C_10_-C_14:1_; Rha-C_8_-C_10_	[67]
*Pseudomonas aeruginosa* NCIM 5514	Bushnell-Hass (BH) medium (pH 7.2) supplemented with 1% (*w*/*v*) glucose		NaCl 15% *w*/*v*		34.61	Rha-C_10_-C_14:1_; Rha-C_8_-C_10_	[67]
*Pseudomonas aeruginosa* NCIM 5514	Bushnell-Hass (BH) medium (pH 7.2) supplemented with 1% (*w*/*v*) glucose		NaCl 16% *w*/*v*		34.82	Rha-C_10_-C_14:1_; Rha-C_8_-C_10_	[67]
*Pseudomonas aeruginosa* NCIM 5514	Bushnell-Hass (BH) medium (pH 7.2) supplemented with 1% (*w*/*v*) glucose		NaCl 18% *w*/*v*		35.50	Rha-C_10_-C_14:1_; Rha-C_8_-C_10_	[67]
*Pseudomonas aeruginosa* NCIM 5514	Bushnell-Hass (BH) medium (pH 7.2) supplemented with 1% (*w*/*v*) glucose		NaCl 20% *w*/*v*		38.57	Rha-C_10_-C_14:1_; Rha-C_8_-C_10_	[67]
*Pseudomonas sp*. MCTG214(3b1)	ZMB supplemented with 1% (*v*/*v*) rapeseed oil				30.13	Rha-Rha-C_10_-C_10_; Rha-Rha-C_10_	[167]
*Pseudomonas aeruginosa* P6	Glucose mineral salts medium (GMSM) + 2% glycerol			200	36	Rha-Rha-C_10_-C_10_; Rha-Rha-C_10_-C_12:1_(Rha-Rha-C_12:1_-C_10_); Rha-Rha-C_12_-C_10_ (Rha-Rha-C_10_-C_12_); Rha-Rha-C_10_-C_8_ (Rha-Rha-C_8_-C_10_)	[75]
*Pseudomonas aeruginosa* AT10	Soybean oil refinery wastes	Water	0	230	27.3	Rha-C_10_-C_10_; Rha-Rha-C_10_-C_12_; Rha-C_10_-C_12_; Rha-C_12:1_-C_10_; Rha-C_12:2_; R_1_C_8:2_	[99]
*Pseudomonas aeruginosa* AT10	Soybean oil refinery wastes	Water	0	150	26.8	Rha-Rha-C_10_C_10_; Rha-C_10_-C_10_; Rha-Rha-C_10_-C_12_; Rha-C_10_-C_12_; Rha-C_12:1_-C_10_; Rha-_1_C_12:2_; Rha-C_8:2_	[99]
*Pseudomonas aeruginosa* mutant MIG-N146	Mineral salts medium (MSM) and 10% (*v*/*v*) corn oil	6.8	NaHCO3 10 mM NaCl	45	28.6	Rha-Rha-C_10_-C_14:1_; Rha- Rha-C_10_-C_12_; Rha-Rha-C_10_-C_12:1_; Rha-Rha-C_10_-C_10_; Rha-Rha-C_10_- C_8_; Rha-Rha-C_8_-C_10_; Rha-Rha-C_10_	[62]
*Pseudomonas aeruginosa* mutant MIG-N146	Mineral salts medium (MSM) and 10% (*v*/*v*) corn oil	6.8	NaHCO_3_ 10 mM NaCl	60	27.6	Rha-Rha-C_10_-C_14:1_; Rha- Rha-C_10_-C_12_; Rha-Rha-C_10_-C_12:1_; Rha-Rha-C_10_-C_10_; Rha-Rha-C_10_- C_8_; Rha-Rha-C_8_-C_10_; Rha-Rha-C_10_	[62]
*Pseudomonas aeruginosa* mutant MIG-N146	Mineral salts medium (MSM) and 10% (*v*/*v*) corn oil	6.8	NaHCO_3_ 10 mM NaCl	120	28.4	Rha-C_14:2_; Rha-C_12:2_; Rha-C_12_-C_10_; Rha- C_10_-C_12:1_; Rha-C_10_-C_10_; Rha-C_10:1_-C_8_; Rha-Rha-C_10_-C_10_	[62]
*Pseudomonas aeruginosa* OBP1	Mineral salts medium (MSM) + 2 g (NH_4_)_2_SO_4_ + 2 g urea + glucose				50.7	Rha-C_10_-C_10_; Rha-Rha-C_10_-C_10_	[72]
*Pseudomonas aeruginosa* OBP1	Mineral salts medium (MSM) + 2 g (NH_4_)_2_SO_4_ + 2 g urea + glycerol				47.2	Rha-C_10_-C_10_; Rha-Rha-C_10_-C_10_	[72]
*Pseudomonas aeruginosa* OBP1	Mineral salts medium (MSM) + 2 g (NH_4_)_2_SO_4_ + 2 g urea + n-hexadecane			45	31.1	Rha-C_10_-C_10_; Rha-Rha-C_10_-C_10_	[72]
*Pseudomonas aeruginosa* OBP1	Mineral salts medium (MSM) + 2 g (NH_4_)_2_SO_4_ + 2 g urea + octadecene				31.9	Rha-C10-C10; Rha-Rha-C10-C10	[72]
*Pseudomonas aeruginosa* OBP1	Mineral salts medium (MSM) + 2 g (NH_4_)_2_SO_4_ + 2 g urea + crude oil				32.7	Rha-C10-C10; Rha-Rha-C10-C10	[72]
*Pseudomonas aeruginosa* OBP1	Mineral salts medium (MSM) + 2 g (NH_4_)_2_SO_4_ + 2 g urea + sunflower oil				37.9	Rha-C10-C10; Rha-Rha-C10-C10	[72]
*Pseudomonas aeruginosa* OBP1	Mineral salts medium (MSM) + 2 g (NH_4_)_2_SO_4_ + 2 g urea + soybean oil				38.3	Rha-C10-C10; Rha-Rha-C10-C10	[72]
*Pseudomonas aeruginosa* strain KVD-HM52	Mineral salts medium (MSM) + molasses 2%			120	33.03	Rha-C10-C10; Rha-Rha-C10-C10	[61]
*Pseudomonas aeruginosa* MM1011	Sunflower oil			120	26	Rha-C10-C10; Rha-Rha-C10-C10	[73]
*Pseudomonas aeruginosa* SG	Glycerol–nitrate (GN) medium		Aerobically	60	27.9	Rha-C_8_-C_10_; Rha-Rha-C_10_-C_12:1_; Rha-Rha-C_8_-C_10_	[70]
*Pseudomonas aeruginosa* SG	Glycerol–nitrate (GN) medium		Anaerobically	80	33.1	Rha-C_10_-C_12_; Rha-C_10_-C_10_	[70]
*Pseudomonas aeruginosa* isolate Bs20	Soybean oil-mineral salts medium (SMSM)			13.4	30	Mono-rhamnolipids 57%; di-rhamnolipids 43%	[64]
*Pseudomonas aeruginosa* isolate Bs20	Soybean oil-mineral salts medium (SMSM)		100 °C		34 ^d^ 34 ^e^ 35 ^f^ 34 ^g^ 34 ^h^ 35 ^i^	Mono-rhamnolipids 57%; di-rhamnolipids 43%	[64]
*Pseudomonas aeruginosa* isolate Bs20	Soybean oil-mineral salts medium (SMSM)		121 °C		35 ^j^	Mono-rhamnolipids 57%; di-rhamnolipids 43%	[64]
*Pseudomonas aeruginosa* isolate Bs20	Soybean oil-mineral salts medium (SMSM)		NaCl 0.3% *w*/*v*		33	Mono-rhamnolipids 57%; di-rhamnolipids 43%	[64]
*Pseudomonas aeruginosa* isolate Bs20	Soybean oil-mineral salts medium (SMSM)		NaCl 0.9% *w*/*v*		35	Mono-rhamnolipids 57%; di-rhamnolipids 43%	[64]
*Pseudomonas aeruginosa* isolate Bs20	Soybean oil-mineral salts medium (SMSM)		NaCl 3% *w*/*v*		35	Mono-rhamnolipids 57%; di-rhamnolipids 43%	[64]
*Pseudomonas aeruginosa* isolate Bs20	Soybean oil-mineral salts medium (SMSM)		NaCl 6% *w*/*v*		35	Mono-rhamnolipids 57%; di-rhamnolipids 43%	[64]
*Pseudomonas aeruginosa* isolate Bs20	Soybean oil-mineral salts medium (SMSM)	2			35	Mono-rhamnolipids 57%; di-rhamnolipids 43%	[64]
*Pseudomonas aeruginosa* isolate Bs20	Soybean oil-mineral salts medium (SMSM)	3			35	Mono-rhamnolipids 57%; di-rhamnolipids 43%	[64]
*Pseudomonas aeruginosa* isolate Bs20	Soybean oil-mineral salts medium (SMSM)	4			35	Mono-rhamnolipids 57%; di-rhamnolipids 43%	[64]
*Pseudomonas aeruginosa* isolate Bs20	Soybean oil-mineral salts medium (SMSM)	5			34	Mono-rhamnolipids 57%; di-rhamnolipids 43%	[64]
*Pseudomonas aeruginosa* isolate Bs20	Soybean oil-mineral salts medium (SMSM)	6			34	Mono-rhamnolipids 57%; di-rhamnolipids 43%	[64]
*Pseudomonas aeruginosa* isolate Bs20	Soybean oil-mineral salts medium (SMSM)	7			32	Mono-rhamnolipids 57%; di-rhamnolipids 43%	[64]
*Pseudomonas aeruginosa* isolate Bs20	Soybean oil-mineral salts medium (SMSM)	8			32	Mono-rhamnolipids 57%; di-rhamnolipids 43%	[64]
*Pseudomonas aeruginosa* isolate Bs20	Soybean oil-mineral salts medium (SMSM)	9			35	Mono-rhamnolipids 57%; di-rhamnolipids 43%	[64]
*Pseudomonas aeruginosa* isolate Bs20	Soybean oil-mineral salts medium (SMSM)	10			36	Mono-rhamnolipids 57%; di-rhamnolipids 43%	[64]
*Pseudomonas aeruginosa* isolate Bs20	Soybean oil-mineral salts medium (SMSM)	11			36	Mono-rhamnolipids 57%; di-rhamnolipids 43%	[64]
*Pseudomonas aeruginosa* isolate Bs20	Soybean oil-mineral salts medium (SMSM)	12			37.5	Mono-rhamnolipids 57%; di-rhamnolipids 43%	[64]
*Pseudomonas aeruginosa* isolate Bs20	Soybean oil-mineral salts medium (SMSM)	13			37.5	Mono-rhamnolipids 57%; di-rhamnolipids 43%	[64]
*Pseudomonas aeruginosa* BN10	Mineral salts medium (MSM) + 2% (*w*/*v*) glucose				33.4	Rha-Rha-C10-C10; Rha-C10-C10	[65]
*Pseudomonas aeruginosa* BN10	Mineral salts medium (MSM) + 2% (*w*/*v*) glycerol			40	27.5	Rha-Rha-C10-C10; Rha-C10-C10	[65]
*Pseudomonas aeruginosa* BN10	Mineral salts medium (MSM) + 2% (*w*/*v*) n-hexadecane				28.3	Rha-Rha-C10-C10; Rha-C10-C10	[65]
*Pseudomonas aeruginosa* BN10	Mineral salts medium (MSM) + 2% (*w*/*v*) n-alkane				70–40.3	Rha-Rha-C10-C10; Rha-C10-C10	[65]
*Pseudomonas aeruginosa* LBI	Soapstock linoleic acid 50%, oleic acid 25%, palmitic acid 7%, and stearic acid 4%			120	24	Rha-Rha-C_10_-C_10_; Rha-Rha-C_10_-C_12:1_; Rha-Rha-C_10_-C_12_; Rha-C_10_-C_12:1_; Rha-C_10_-C_12_	[66]
*Pseudomonas aeruginosa* LBI	Mineral salts medium (MSM) + 2% (*w*/*v*) glucose				35.76	Rha-Rha-C_10_-C_10_	[69]
*Pseudomonas aeruginosa* LBI	Mineral salts medium (MSM) + 2% (*w*/*v*) glycerol				34.80	Rha-Rha-C_10_-C_10_	[69]
*Pseudomonas aeruginosa* LBI	Mineral salts medium (MSM) + 2% (*w*/*v*) used soybean oil				30.80	Rha-C_10_-C_10_	[69]
*Pseudomonas aeruginosa* LBI	Mineral salts medium (MSM) + 2% (*w*/*v*) chicken fat				32.76	Rha-C_10_-C_10_	[69]
*Pseudomonas aeruginosa* LBI	Mineral salts medium (MSM) + 2% (*w*/*v*) soybean oil soapstock				32.36	Rha-C_10_-C_10_	[69]
*Pseudomonas aeruginosa* LBI	Mineral salts medium (MSM) + 2% (*w*/*v*) cottonseed oil waste			86.79	33.86	Rha-C_10_-C_10_	[69]
*Pseudomonas aeruginosa* LBI	Mineral salts medium (MSM) + 2% (*w*/*v*) babassu oil waste			210.77	30.08	Rha-C_10_-C_10_	[69]
*Pseudomonas aeruginosa* LBI	Mineral salts medium (MSM) + 2% (*w*/*v*) corn oil waste			43.21	30.96	Rha-C_10_-C_10_	[69]
*Pseudomonas aeruginosa* LBI	Soybean oil waste			51.56	26.92	Rha-C_10_-C_10_	[69]
*Pseudomonas aeruginosa* LBI	Palm oil waste			40.19	31.76	Rha-C_10_-C_10_	[69]
*Pseudomonas aeruginosa* SP4	Nutrient broth + 2% (*w*/*v*) inoculum + 2% (*w*/*v*) palm oil	PBS pH 7.4	0	200	29	Rha-C_10_-C_10_	[74]
*Pseudomonas aeruginosa* SP4	Nutrient broth + 2% (*w*/*v*) inoculum + 2% (*w*/*v*) palm oil	PBS pH 7.4	0.1 M NaCl	200	29	Rha-C_10_-C_10_	[74]
*Pseudomonas aeruginosa* SP4	Nutrient broth + 2% (*w*/*v*) inoculum + 2% (*w*/*v*) palm oil	PBS pH 7.4	0.2 M NaCl	200	29	Rha-C_10_-C_10_	[74]
*Pseudomonas aeruginosa* SP4	Nutrient broth + 2% (*w*/*v*) inoculum + 2% (*w*/*v*) palm oil	PBS pH 7.4	0.4 M NaCl	200	29	Rha-C_10_-C_10_	[74]
*Pseudomonas aeruginosa* SP4	Nutrient broth + 2% (*w*/*v*) inoculum + 2% (*w*/*v*) palm oil	PBS pH 7.4	0.1 M EtOH	300	29	Rha-C_10_-C_10_	[74]
*Pseudomonas aeruginosa* SP4	Nutrient broth + 2% (*w*/*v*) inoculum + 2% (*w*/*v*) palm oil	PBS pH 7.4	0.2 M EtOH	600	29	Rha-C_10_-C_10_	[74]
*Pseudomonas aeruginosa* SP4	Nutrient broth + 2% (*w*/*v*) inoculum + 2% (*w*/*v*) palm oil	PBS pH 7.4	0.4 M EtOH	600	29	Rha-C_10_-C_10_	[74]
*Pseudomonas aeruginosa* CCTCC AB93066	Mineral salts medium (MSM) + 20 g/L glucose	6.5		35	32	Rha-C10-C10 Rha-C10-C12–H2 Rha-C10-C12	[58]
*Pseudomonas aeruginosa* CCTCC AB93066	Mineral salts medium (MSM) + 20 g/L glucose	6.5		70	36	Rha-Rha-C10-C10 Rha-Rha-C10-C12–H2 Rha-Rha-C10-C12	[58]
*Pseudomonas* sp. pyr 41	Proteose peptone glucose ammonium salt (PPGAS) medium + glycerol			100	31.93	Di-rhamnolipids	[154]
*Pseudomonas aeruginosa* LCD12	Proteose peptone glucose ammonium salt (PPGAS) medium + glycerol			50	29.85	Mono-rhamnolipids ~50%; di-rhamnolipids ~50%	[154]
*Pseudomonas aeruginosa* D2	Proteose peptone glucose ammonium salt (PPGAS) medium + glycerol			80	31.27	Di-rhamnolipids	[154]
*Pseudomonas aeruginosa* PAO1	Proteose peptone glucose ammonium salt (PPGAS) medium + glycerol			60	31.23	Di-rhamnolipids	[154]
*Pseudomonas aeruginosa* PG201	Glycerol 2% (*w*/*v*) + hexadecane 1% (*w*/*v*)				25.96	Mono-rhamnolipids: di-rhamnolipids 2:1	[168]
*Pseudomonas stutzeri* Rhl	Luria–Bertani (LB) medium + glycerol			60			[68]
*Pseudomonas stutzeri* Rhl	Luria–Bertani (LB) medium + glycerol			90	30.3 ^k^		[68]
*Pseudomonas stutzeri* Rhl	Luria–Bertani (LB) medium + glycerol	pH 2–8			32		[68]
*Pseudomonas stutzeri* Rhl	Luria–Bertani (LB) medium + glycerol		NaCl 0–18%		31.5		[68]
*Burkholderia thailandensis* E264	Nutrient broth + 4% (*w*/*v*) glycerol	pH 7	Water	125	30	Mono-rhamnolipids: di-rhamnolipids 1:3	[88]
*Marinobacter* sp. MCTG107b	ZM/1 medium supplemented with 1% (*v*/*v*) rapeseed oil 1% (*w*/*v*) glucose				31	Rha-Rha-C_10_-C_10_ Methyl-Rha-Rha-C_10_-C_10_	[169]

^a^ 1h incubation. ^b^ 25 h incubation. ^c^ 120 h incubation. ^d^ 0 min incubation. ^e^ 5 min incubation. ^f^ 10 min incubation. ^g^ 20 min incubation. ^h^ 40 min incubation. ^i^ 60 min incubation. ^j^ 10 min incubation. ^k^ Temperature ranging between 25 and 120 °C.

**Table 3:** The *cmc* and surface properties of “pure” rhamnolipids (obtained either via purification of microbial extracts or via modified biosynthetic routes) and synthetic rhamnolipids. According to the rhamnolipid pKa = 5.5, rhamnolipids are expected to be neutral at pH < 5.5 and negatively charged at pH > 5.5.

Rhamnolipid	pH	Additive	Purity	*cmc* (mmol/L)	*γ_min_* (mN/m)	*A_min_* (Å^2^)	*n*	Ref
**Rha-C_10_-C_10_ (R1)**	2	-	purified		27.5			[170]
	4	-	purified		27.5			[170]
	4	Sodium citrate 100 mM	95 ^†^	0.04				[71]
	4	Sodium citrate 100 mM NaCl 0.05 M	95 ^†^	0.04				[71]
	4	Sodium citrate 100 mM NaCl 0.1 M	95 ^†^	0.05	30	63.2		[71]
	4	Sodium citrate 100 mM NaCl 0.2 M	95 ^†^	0.04				[71]
	4	Sodium citrate 100 mM NaCl 0.3 M	95 ^†^	0.05				[71]
	4	Sodium citrate 100 mM NaCl 0.5 M	95 ^†^	0.05				[71]
	4	Sodium citrate 100 mM NaCl 1 M	95 ^†^	0.04				[71]
	5	Sodium acetate buffer	96	0.04	28.2	59.3	1	[171]
	6	-	purified		27.5			[170]
	6.5	-	80	0.075	32			[58]
	6.8	-	96	0.1	30/30	135.1	2	[171]
	6.8	-	96	0.1	30	135.1	2	[29]
	6.8	NaCl 0.05 M	96	0.1	28.6	71.4	1	[172]
	6.8	NaCl 0.5 M	96	0.05	28.5	66.1	1	[172]
	6.8	NaCl 1.0 M	96	0.04	28.6	83.6	1	[172]
	6.8	-	96	0.1	30	68	1	[172]
	6.8	-	purified	0.40	27.44	110	2	[170]
	6.8	NaCl 0.3 M	purified	0.20	27.18			[170]
	6.8	NaCl 0.6 M	purified	0.16	27.00			[170]
	6.8	NaCl 1.4 M	purified	0.16	27.19			[170]
	6.8	NaCl 1.7 M	purified	0.16	27.29			[170]
	6.8	Rhamnose (rhamnose: R1 = 1)	99	0.07	28.6			[173]
	6.8	-	98 ^††^	0.108	25.2	98	2	[174]
	7	KH_2_PO_4_ 0.063 M NaOH 0.037 M	98 ^††^	0.130	26.3	109	2	[174]
	7	KH_2_PO_4_ 0.063 M NaOH 0.037 M	purified	0.18	28.7	66	1	[175]
	7	-	purified		27.5			[170]
	7.4	Hepes 5 mM	95 ^†^	0.25				[71]
	7.4	Hepes 5 mM NaCl 0.05 M	95 ^†^	0.09				[71]
	7.4	Hepes 5 mM NaCl 0.1 M	95 ^†^	0.07	36	57.2	1	[71]
	7.4	Hepes 5 mM NaCl 0.2 M	95 ^†^	0.06				[71]
	7.4	Hepes 5 mM NaCl 0.3 M	95 ^†^	0.05				[71]
	7.4	Hepes 5 mM NaCl 0.5 M	95 ^†^	0.04				[71]
	7.4	Hepes 5 mM NaCl 1 M	95 ^†^	0.06				[71]
	8	-	purified		27.4			[170]
	8	Phosphate buffer 10 mM	98 ^††^	0.201	29.0	86	2	[174]
	9	Borax 0.023 M HCl 0.008 M	purified	0.36	31.2	77	1	[175]
	9	Borax 0.023 M HCl 0.008 M	purified	0.36	31.2	75		[176]
	10	-	purified		27.4			[170]
	12	-	purified		31			[170]
	*	-	purified	0.04	27.9	53	1	[175]
	*	NaCl 0.5 M	purified	0.03	27.8	52	1	[165]
	*	-	95 ^†††^	1.17 ^a^	-	-	-	[144]
	*	NaCl 0.8 M	95 ^†††^	0.22 ^a^	-	-	-	[144]
	*	-	95 ^†††^	0.052 ^b^ 0.048 ^c^ 0.051 ^d^ 0.050 ^e^	27.89	82.6	2	[177]
**R,R**	4	-	pure	0.016	27.5	21	1	[147]
**R,S**	4	-	pure	0.025	28.8	23	1	[147]
**S,S**	4	-	pure	0.018	27.5	21	1	[147]
**S,R**	4	-	pure	0.015	28.2	21	1	[147]
**R,R**	8	-	pure	0.27	28.1	117	2	[147]
**R,S**	8	-	pure	0.079	27.4	80	2	[147]
**S,S**	8	-	pure	0.201	29.5	93	2	[147]
**S,R**	8	-	pure	0.180	28.5	103	2	[147]
**Rha-C_14_**	8	-	-	1.646	31.67	88.96	2	[148]
**Rha-C_14_-C_6_**	8	-	96	0.519	35.97	113.87	2	[148]
**Rha-C_14_-C_8_**	8	-	95	0.02977	25.83	61.78	2	[148]
**Rha-C_14_-C_10_**	8	-	96	0.01034	23.84	37.78	2	[148]
**Rha-C_14_-C_12_**	8	-	97	0.05275	26.26	63.01	2	[148]
**Rha-C_14_-C_14_**	8	-	98	0.15265	35.96	48.78	2	[148]
**Rha-Rha-C_10_-C_10_ (R2)**	2	-	purified		29.5			[170]
	4	-	purified		34			[170]
	4	Sodium citrate 100 mM	purified	0.01				[59]
	4	Sodium citrate 100 mM NaCl 0.1 M	50 ^††††^	0.01		76	1	[59]
	4	Sodium citrate 100 mM NaCl 0.2 M	50 ^††††^	0.01				[59]
	4	Sodium citrate 100 mM NaCl 0.5 M	50 ^††††^	0.01				[59]
	5	Sodium acetate buffer	50 ^††††^	0.04	28.2	64.8	1	[171]
	6	-	purified		34			[170]
	6	Phosphate-buffered saline (PBS)	70	0.062				[178]
	6.5	Phosphate-buffered saline (PBS)	70	0.078				[178]
	6.5	-	70	0.106	35			[58]
	6.8	-	99	0.15	31.2	131.0	2	[171]
	6.8	-	99	0.15	31.2	131	2	[172]
	6.8	NaCl 0.05 M	99	0.08	31.2	67.3	1	[172]
	6.8	NaCl 0.5 M	99	0.08	31.2	68.2	1	[172]
	6.8	NaCl 1.0 M	99	0.04	31.0	79.8	1	[172]
	6.8	-	99	0.15	30	56	1	[179]
	6.8	-	purified	0.46	31.27	138	2	[170]
	6.8	NaCl 0.3 M	purified	0.15	29.36			[170]
	6.8	NaCl 0.6 M	purified	0.09	29.79			[170]
	6.8	NaCl 1.4 M	purified	0.09	29.67			[170]
	6.8	NaCl 1.7 M	purified	0.08	29.71			[170]
	6.8	-	99	0.15	32	131	2	[173]
	6.8	Rhamnose (rhamnose: R2 = 1)	99	0.05	34		2	[173]
	7	Phosphate-buffered saline (PBS)	70	0.082				[178]
	7	-	purified		33.5			[170]
	7	KH_2_PO_4_ 0.063 M NaOH 0.037 M	purified	0.11	34.7	77	1	[175]
	7.4	Hepes 5 mM NaCl 0 M	50 ^††††^	0.5				[59]
	7.4	Hepes 5 mM NaCl 0.075 M	50 ^††††^	0.18				[59]
	7.4	Hepes 5 mM NaCl 0.15 M	50 ^††††^	0.11		76	1	[59]
	7.4	Hepes 5 mM NaCl 0.3 M	50 ^††††^	0.11				[59]
	7.4	Hepes 5 mM NaCl 0.5 M	50 ^††††^	0.1				[59]
	7.5	Phosphate-buffered saline (PBS)	70	0.083				[178]
	8	Phosphate-buffered saline (PBS)	70	0.082				[178]
	8	-	purified		34			[170]
	9	Borax 0.023 M HCl 0.008 M	purified	0.18	37.4	80	1	[175]
	9	Borax 0.023 M HCl 0.008 M	purified	0.18	37.4	79	1	[176]
	10	-	purified		33			[170]
	12	-	purified		35.5			[170]
	*	-	purified	0.07	30.3	84	1	[175]
	*	NaCl 0.5 M	purified	0.08	30.4	79	1	[175]
	*	-	purified	0.17 **	28.8	100 ***	2	[99]
	*		purified	0.315	28			[180]
	*	-	95	0.97 ^e^				[144]
	*	NaCl 0.6 M	95	0.17 ^e^				[144]
**Methyl-Rha-Rha-C10-C10**	*		purified	0.495 ^f^	30.6			[180]

^†^ Rha-C_10_-C_10_, Rha-C_10_-C_12_ and Rha-C_10_-C_12:1_. ^††^ Mono-rhamnolipids 98%, Rha-C_10_-C_10_ 72%. ^†††^ Commercial product mainly composed of Rha-C_10_-C_10_ (95% rhamnolipids of which 90% mono-rhamnolipids). ^††††^ Rha-Rha-C_10_-C_10_ ~50%, Rha-Rha-C_10_-C_12_ ~29%. ^a^ Calculated by fluorescence titration using pyrene as a probe. ^b^ Determined by following surface tension changes, calculated from *cmc* (mg/L), taking into account a molecular weight of 504 Da. ^c^ Determined by following density changes, calculated from *cmc* (mg/L), taking into account a molecular weight of 504 Da. ^d^ Determined by following viscosity changes, calculated from *cmc* (mg/L), taking into account a molecular weight of 504 Da. ^e^ Determined by following conductivity changes, calculated from *cmc* (mg/L), taking into account a molecular weight of 504 Da. ^f^ Calculated by fluorescence titration using ANS (8-Anilinonaphthalene-1-sulfonic acid) as a probe. * Water solutions, the pH was not measured. ** Calculated from *cmc* (mg/L), taking into account a molecular weight of 650 Da. *** Calculated from maximum surface excess, taking into account a molecular weight of 650 Da.
